# RV144 HIV-1 vaccination impacts post-infection antibody responses

**DOI:** 10.1371/journal.ppat.1009101

**Published:** 2020-12-08

**Authors:** Thembi Mdluli, Ningbo Jian, Bonnie Slike, Dominic Paquin-Proulx, Gina Donofrio, Aljawharah Alrubayyi, Syna Gift, Rebecca Grande, Mary Bryson, Anna Lee, Vincent Dussupt, Letzibeth Mendez-Riveria, Eric Sanders-Buell, Agnès-Laurence Chenine, Ursula Tran, Yifan Li, Eric Brown, Paul T. Edlefsen, Robert O’Connell, Peter Gilbert, Sorachai Nitayaphan, Punnee Pitisuttihum, Supachai Rerks-Ngarm, Merlin L. Robb, Robert Gramzinski, Galit Alter, Sodsai Tovanabutra, Ivelin S. Georgiev, Margaret E. Ackerman, Victoria R. Polonis, Sandhya Vasan, Nelson L. Michael, Jerome H. Kim, Michael A. Eller, Shelly J. Krebs, Morgane Rolland

**Affiliations:** 1 U.S. Military HIV Research Program, Walter Reed Army Institute of Research, Silver Spring, Maryland, United States of America; 2 Henry M. Jackson Foundation for the Advancement of Military Medicine, Bethesda, Maryland, United States of America; 3 Dartmouth College, Hanover, New Hampshire, United States of America; 4 SCHARP, Seattle, Washington, United States of America; 5 AFRIMS, Bangkok, Thailand; 6 Mahidol University, Bangkok, Thailand; 7 Thai Ministry of Public Health, Nonthaburi, Thailand; 8 Ragon Institute, Boston, Massachusetts, United States of America; 9 Vanderbilt Vaccine Center, Vanderbilt University Medical Center, Nashville, Tennessee, United States of America; Vaccine Research Center, UNITED STATES

## Abstract

The RV144 vaccine efficacy clinical trial showed a reduction in HIV-1 infections by 31%. Vaccine efficacy was associated with stronger binding antibody responses to the HIV Envelope (Env) V1V2 region, with decreased efficacy as responses wane. High levels of Ab-dependent cellular cytotoxicity (ADCC) together with low plasma levels of Env-specific IgA also correlated with decreased infection risk. We investigated whether B cell priming from RV144 vaccination impacted functional antibody responses to HIV-1 following infection. Antibody responses were assessed in 37 vaccine and 63 placebo recipients at 6, 12, and 36 months following HIV diagnosis. The magnitude, specificity, dynamics, subclass recognition and distribution of the binding antibody response following infection were different in RV144 vaccine recipients compared to placebo recipients. Vaccine recipients demonstrated increased IgG1 binding specifically to V1V2, as well as increased IgG2 and IgG4 but decreased IgG3 to HIV-1 Env. No difference in IgA binding to HIV-1 Env was detected between the vaccine and placebo recipients following infection. RV144 vaccination limited the development of broadly neutralizing antibodies post-infection, but enhanced Fc-mediated effector functions indicating B cell priming by RV144 vaccination impacted downstream antibody function. However, these functional responses were not associated with clinical markers of disease progression. These data reveal that RV144 vaccination primed B cells towards specific binding and functional antibody responses following HIV-1 infection.

## Author summary

The RV144 vaccine efficacy trial showed a reduction in HIV-1 infections that associated with binding antibody responses to the Envelope (Env) V1V2 loops but precise mechanisms remain unclear. To evaluate the effect of vaccine priming, we performed a systems serology analysis in 37 vaccine and 63 placebo recipients 6, 12 and 36 months after HIV-1 breakthrough infections. Vaccinees were characterized by strong V1V2-specific antibody responses synergized with V1V2-specific ADCP responses, whereas placebo recipients had stronger IgG3 and gp120-specific responses. The strongest distinguishing feature for vaccinees was IgG4 responses. RV144 vaccination enhanced Fc-mediated effector functions but limited the development of broadly neutralizing antibodies post-infection, which were found in eight placebo recipients but no vaccinee. These data show that RV144 vaccination primed B cells towards specific binding and functional antibody responses, with differences between groups still manifest three years after infection, i.e. on average five years after vaccination. These long-term consequences highlight that imprinting certain functions (while deterring other responses) could offer benefits even for leaky vaccines.

## Introduction

Despite advances in prophylaxis and treatment, an HIV-1 vaccine is needed to curb the AIDS epidemic [1]. The RV144 HIV-1 vaccine efficacy trial showed a reduction in HIV-1 infections, 59.9% at 1 year and 31.2% at 3.5 years post-vaccination [2]. RV144 enrolled 16,402 Thai participants who received either a vaccine or a placebo. The vaccine regimen consisted of a canarypox vector prime (ALVAC encoding subtype B gag/pro inserts and a CRF01_AE Envelope (Env) insert) given at 0, 1, 3 and 6 months, accompanied by two recombinant gp120 proteins (AIDSVAX: subtype B MN and CRF01_AE A244) given at 3 and 6 months. A correlates study showed that the decreased risk of HIV-1 infection was associated with IgG binding antibodies (Ab) targeting the variable loops V1 and V2 of the HIV-1 Env glycoprotein (V1V2) [3,4]. After responses peaked at 26 weeks (measured two weeks after the last immunization), V1V2 binding antibodies waned rapidly over time, which associated with waning efficacy. Notably, the presence of V1V2 binding antibodies and functional antibodies to HIV-1 Env was low or absent 6 months following vaccination in individuals who became infected. The importance of V2-specific responses was corroborated by a sieve analysis which showed that breakthrough sequences from vaccine recipients differed from those of placebo recipients at amino acid (AA) sites 169 and 181 in V1V2 [5]. In addition, low Env-specific IgA responses or a high IgG/IgA ratio combined with high ADCC responses were identified as a secondary correlate of decreased risk of infection [3]. These results highlighted the importance of binding antibodies in the effect of RV144, prompting further research into non-neutralizing Fc-mediated functions.

Comparison of ALVAC/AIDSVAX (RV144) to administration of AIDSVAX alone (VAX003) revealed the importance of the combined regimen [6]. The RV144 regimen showed that IgG3 antibodies to V1V2 were correlated with a decreased risk of infection. High IgG3 responses also correlated with high ADCC responses suggesting that protection was mediated via Fc effector functional activity. These IgG3 antibodies were not durable; response rates declined from 79% to 0% between peak immunity (week 26) and six months later (week 52), correlating with a decrease in vaccine efficacy. A similar study confirmed that elevated gp120-specific IgG3 responses, relative to other features, distinguished the RV144 vaccine antibody profile [7]. IgG3 antibodies against V1V2 were proposed to work in conjunction with IgG1 to induce ADCC and ADCP in RV144, but not in VAX003 [8]. IgG1 and IgG3 responses are considered conducive to favorable humoral responses, whereas IgG2 and particularly IgG4 can dampen humoral responses [9]. For VAX003, the high proportion of IgG4 responses was deemed characteristic of a non-efficacious vaccine profile [8].

In contrast with the role of Fc-mediated effector functions in the RV144-induced protection [6–8], only weak neutralizing antibodies were elicited by the RV144 vaccine, which were not linked with vaccine efficacy [3]. Cross-reactive NAbs arise during HIV-1 infection: approximately 20% of HIV-infected individuals produce NAbs with high breadth and potency [10–12]. The isolation of broad neutralizing antibodies from these individuals illustrates the wide range of targets on the HIV-1 Env, including different V2 apex bnAbs such as PG9, PGDM1400, and CAP256-VRC26 [13–15]. Therefore, since the RV144 vaccine regimen primed B cell responses to V1V2, we wanted to determine whether the development of neutralizing antibodies and Fc-mediated effector functions to V1V2 was accelerated in RV144-vaccinated individuals compared to placebo recipients following infection.

Here we investigated the impact of vaccination on binding and functional antibody responses following HIV-1 breakthrough infection in RV144 vaccine and placebo recipients. Despite priming antibody responses to V1V2, RV144 vaccination restricted the development of broadly neutralizing antibodies, while Fc-mediated effector functions, especially V1V2-specific ADCP, were increased in vaccine recipients who became infected. This study highlights the impact of vaccination on downstream functional antibody responses in individuals with breakthrough infections.

## Results

### RV144 vaccination altered antibody binding specificity, magnitude, dynamics and subclass recognition and distribution to HIV-1 following infection

**Fig 1:**
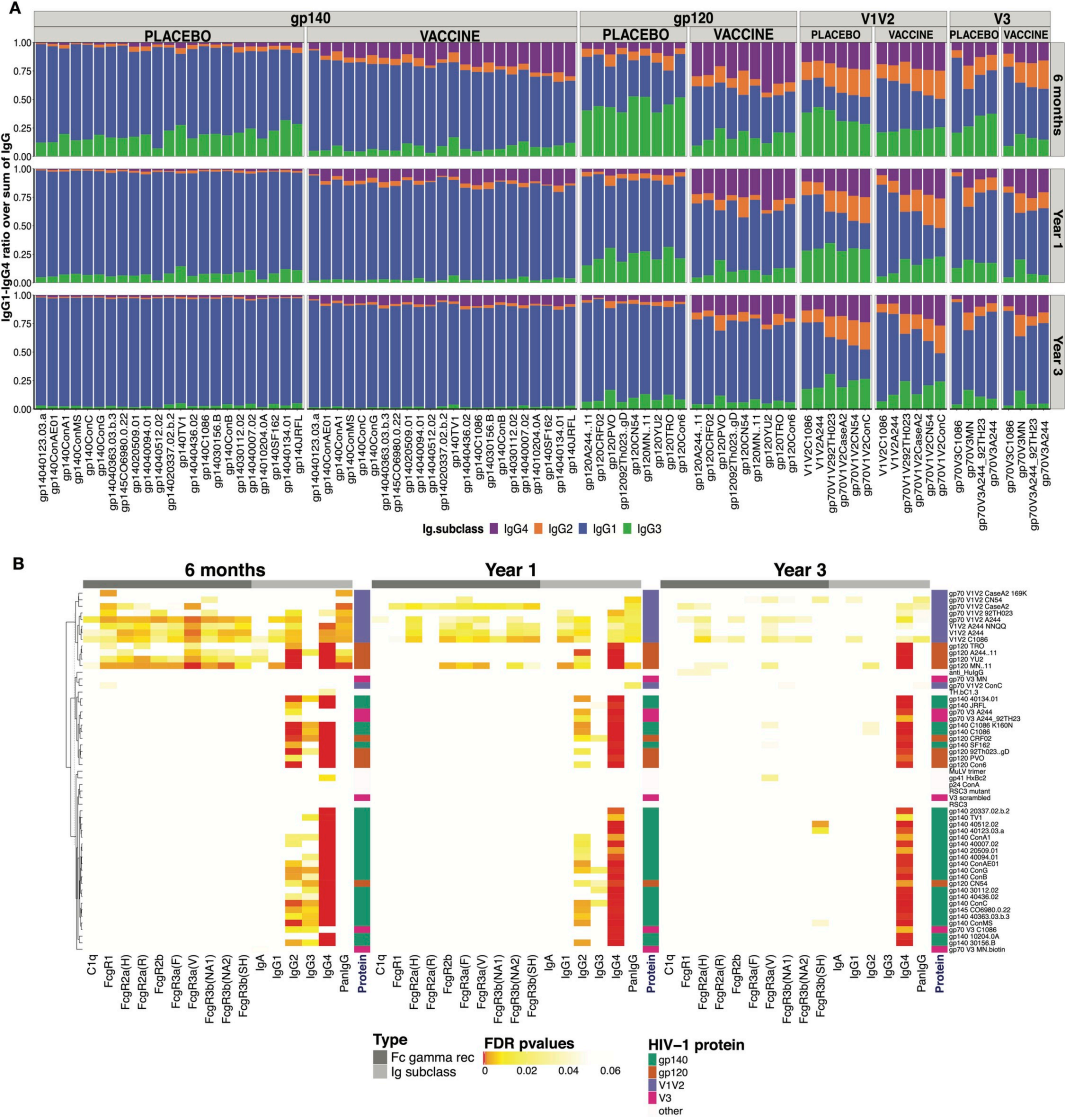
RV144 vaccination primed IgG2, IgG4 and V1V2 binding responses after HIV-1 breakthrough infection. **(**A) Distribution of IgG subclasses to HIV-1 antigens in vaccine and placebo recipients at 6 months (N = 24 vaccine, 31 placebo), one year (N = 18 vaccine, 39 placebo), and three years (N = 27 vaccine, 49 placebo) after HIV-1 diagnosis. Mean signal of antigen specific IgG1, IgG2, IgG3 and IgG4 (blue, orange, green, purple, respectively) are represented as a fraction of the sum of all IgG subclasses. (B) Significant differences between vaccine and placebo groups, using Mann–Whitney U test, measured at 6 months, 1 and 3 years after diagnosis are represented with colored cells. Each cell represents FDR-adjusted p-values where the color indicates the statistical significance: FDR ≤ 0.001 in red, FDR ≤ 0.01 in orange, FDR ≤ 0.05 in yellow and white marks non-significant differences. Columns represent binding responses against complement (C1q), FcγRs, Ig isotypes and subclasses. Each row corresponds to an HIV-1 antigen: gp140 (green), gp120 (orange), V1V2 (purple) and V3 (magenta). The axis on the right represents the hierarchy of clusters.

To determine the effect of B cell priming by RV144 vaccination on post-infection antibody responses, we characterized responses from 37 vaccine and 63 placebo participants who had received all immunizations (2 ALVAC and 2 ALVAC+AIDSVAX) among the 110 who became infected with HIV-1 CRF01AE viruses. Antibody profiling [16] on 188 samples assessed binding antibody subclass, specificity and magnitude against 52 HIV-1 antigens (24 gp140, 9 gp120, 9 V1V2, 5 V3, 5 other HIV-specific antigens) that included antigens used in the RV144 correlates analysis [3] and 16 CRF01_AE antigens (**S1 Table**). We compared immune responses from vaccine and placebo samples collected at three time points after HIV-1 diagnosis: 6 months (29–266 days; 29 vaccine, 31 placebo), 1 year (295–454 days; 18 vaccine, 39 placebo) and 3 years post-diagnosis (805–1188 days; 27 vaccine, 49 placebo). The distribution of IgG subclasses showed that IgG1 binding antibodies targeting gp140 and gp120 Envs dominated in both vaccine and placebo recipients **(Fig 1A)**. Vaccine recipients showed more IgG2 and IgG4 but less IgG3 binding antibodies than placebo recipients for gp120 and gp140 HIV-1 antigens. The IgG subclass distribution for V1V2 or V3 antigens was more balanced than the distribution seen for gp140 and gp120 antigens **(Fig 1A).** The dynamics of the binding antibody response from 6 to 36 months following infection (**S1 Fig**) showed significant increases in the proportion of total IgG and IgG1 to Env overtime. The proportion of IgG2 and IgG4 responses decreased over time and specifically between 6 and 12 months after diagnosis. IgG3 responses also declined over time with a steeper decrease between 6 and 12 months after diagnosis particularly among placebo recipients (**S1 Fig)**.

**Fig 2:**
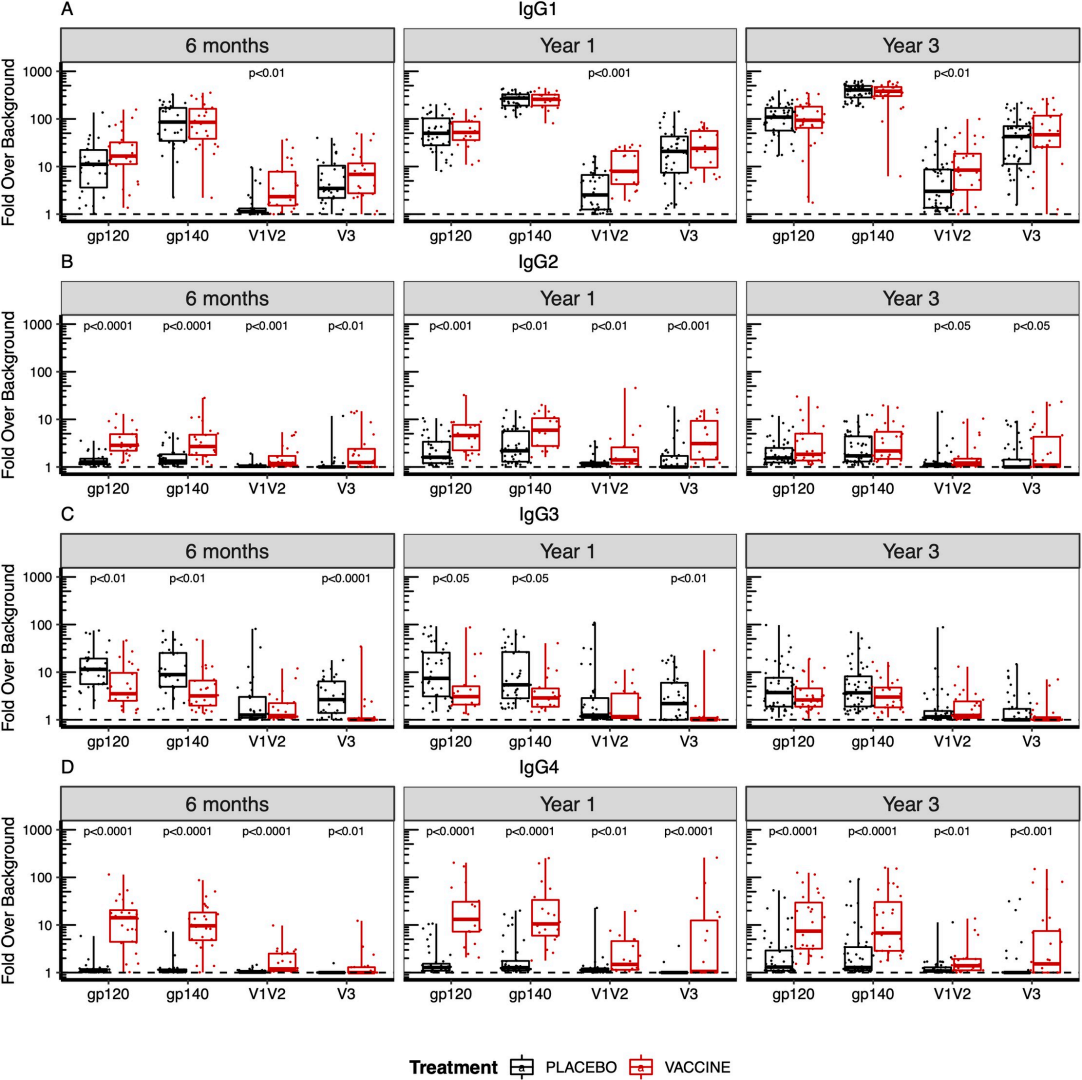
B cell priming in vaccine recipients altered subclass and antibody specificity to HIV-1 following infection. (A) IgG1, (B) IgG2, (C) IgG3, and (D) IgG4 subclass binding to HIV-1 Env antigens at 6 months (N = 24 vaccine, 31 placebo), one year (N = 18 vaccine, 39 placebo), and three years (N = 27 vaccine, 49 placebo) post-HIV-1 diagnosis. The fold over background is represented for each Env antigen as a composite score corresponding to the geometric mean of the fold over background across the different antigens tested: 9 gp120s, 23 gp140s, 6 V1V2 gp70 and 4 V3 gp70 antigen. P values were calculated by Mann-Whitney U test and adjusted by False Discovery Rate (FDR) for multiple comparisons. Only significant (p<0.05) p values are shown.

Next, we compared responses across treatment groups (**Figs 1B and 2**). RV144 vaccine recipients had significantly higher levels of total IgG and IgG1, IgG2 and IgG4 binding antibodies to V1V2 compared to placebo recipients at 6, 12 and 36 months post-diagnosis, indicating that the RV144 vaccine primed B cells towards V1V2 (**Figs 1B, 2 and S2**). Compared to placebo recipients, significantly higher levels of IgG2 binding antibodies were detected to all HIV-1 antigens 6 and 12 months following diagnosis in the vaccine recipients (**Figs 1B and 2B)**. Vaccine recipients showed significantly higher IgG4 binding antibodies to all antigens compared to placebo recipients, this persisted until three years after diagnosis (**Figs 1B and 2D**). Contrary to the other subclasses, six months after diagnosis, the RV144 vaccine recipients showed significantly lower levels of IgG3 binding antibodies to gp120 and gp140 Envs and V3, but not to V1V2 compared to the placebo recipients (**Figs 1B and 2C**). The vaccine/placebo difference in HIV-specific IgG3 binding antibodies waned over time and there was no difference between groups by year 3 (**Figs 1B and 2C**). IgA responses, which had been associated with an increased risk of infection in the RV144 correlates analysis, were low and did not differ between treatment groups (**Figs 1B and S2**). In addition, Fcγ receptor engagement of binding antibodies targeting the HIV-1 Env were evaluated using 9 Fcγ receptors (**Fig 1B)**. Significant differences in Fcγ receptor engagement were observed for binding antibodies targeting the V1V2 region between vaccine and placebo recipients, likely due to dominant IgG1 responses (**Fig 1A**).

### The feature that best distinguished vaccine from placebo recipients was IgG4 responses

To assess which Ab binding features most clearly distinguished vaccination groups, we performed feature selection and discriminatory analyses. We filtered redundant immune features to reduce the number of variables to 221, 286 and 284 at 6 months, year 1 and year 3, respectively. From this subset, we found that an IgG4 gp120/gp140 response was consistently selected as the most predictive feature of the vaccine group by analyzing variable importance through Random Forest (**S3 Fig**). As the high predictive power of IgG4 binding antibodies targeting gp120 or gp140 could mask the effect of other predictors, we performed Random Forest separately for each HIV-1 antigen. Interestingly, for V1V2, more features (instead of a single gp120/gp140 feature) had importance scores above 70% (**S3 Fig**).

**Fig 3:**
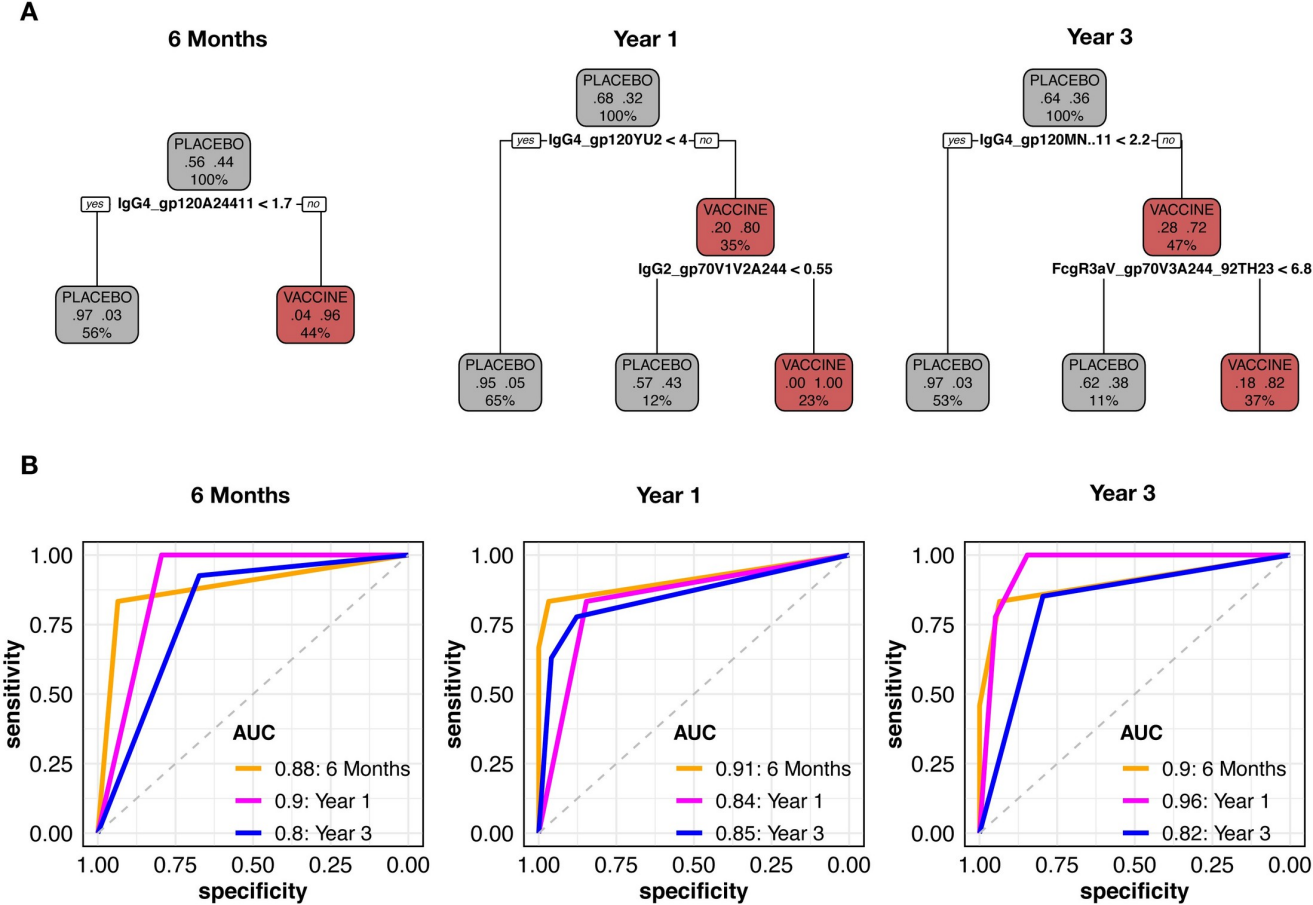
IgG4 gp120 features were the most informative to classify the vaccine group. (A) Representative CART trees generated with the top non-redundant predictors which classified vaccine (red) and placebo recipients (black). (B) ROC curves were generated for CART models corresponding to each time point and tested with data from the other time points. AUC generated with the data used to train the model were evaluated through cross-validation while AUC at other time points (time points with data that were not used to train the model) were evaluated by testing the model with these data.

Using these selected predictors, we generated exploratory CART trees: at each time point, an IgG4 gp120 response was the most informative feature to classify vaccine and placebo participants **(Fig 3**). At 6 months, the IgG4 to A244Δ11 gp120 provided the best split, classifying 91% of the lower responders as placebo recipients and 96% of the high responders as vaccine recipients. The performance of the model was tested through cross-validation with the training data from month 6, achieving an AUC of 0.88 as well as with separate data from year 1 and year 3, achieving AUCs of 0.9 and 0.8, respectively. Data from years 1 and 3 replicated that a gp120-specific IgG4 response was the most discriminating (year 1: IgG4.gp120YU2 and year 3: IgG4.gp120MNΔ11) (**Fig 3**). In addition, a V1V2-specific IgG2 response could further split the treatment groups at year 1, but by year 3, the difference between vaccine and placebo recipients had waned (**Fig 3**). Receiver operator curves (ROC) showed that the CART models performed well (AUC above 0.8) on both training and testing data, showing the lasting effect of the IgG4 priming up to 3 years post-diagnosis. LASSO models confirmed this robust selection of IgG4 responses from 6 months up to year 3 post-diagnosis (**S4 Fig**). Treatment groups were separated into responders (for vaccine recipients) and non-responders (for placebo recipients), using the top two selected immune predictors at all time points (scatter plots in **S4 Fig**). The performance of LASSO models was validated through permutation tests and trained models achieved a significantly higher balanced accuracy compared to group-permutated null models (**S4 Fig**).

### RV144 vaccination restricted the development of broadly neutralizing antibodies following infection

**Fig 4:**
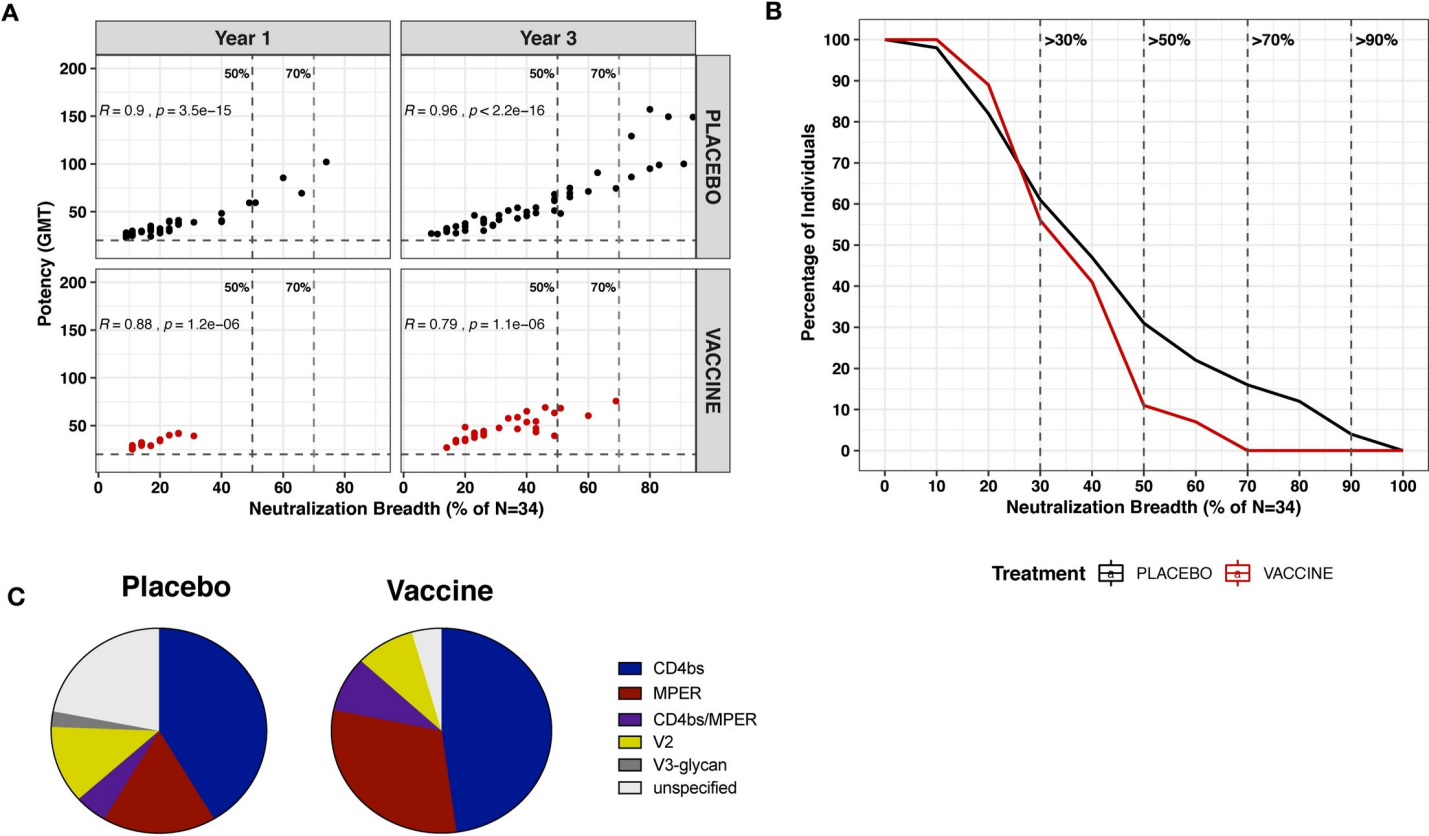
RV144 vaccination restricted the development of broadly neutralizing antibodies following HIV-1 infection. Vaccine recipients are represented in red and placebo recipients in black. (A) Neutralization breadth and potency (geometric mean titer (GMT)) of vaccine and placebo recipients at year 1 (N = 18 vaccine, 39 placebo) and year 3 (N = 27 vaccine, 49 placebo). Neutralization breadth is the percentage of viruses neutralized out of 34 pseudoviruses. Vertical lines indicate 50% and 70% neutralization breadth. (B) Reverse cumulative distribution curves showing the proportion of vaccine (N = 27) and placebo recipients (N = 49) who neutralized a specific fraction of the panel of 34 pseudoviruses at year 3 post-diagnosis. Only infected placebo recipients achieved >70% neutralization breadth (N = 8) at year 3 post-diagnosis. (C) Predicted neutralization specificity of vaccine and placebo recipients with >30% neutralization breadth.

Since increased V1V2 binding responses were observed in the vaccine recipients, we next wanted to determine if B cell priming from RV144 vaccination accelerated the production of broadly neutralizing antibodies targeting V2. One hundred and eighty samples at 12- and 36- months following HIV-1 diagnosis and prior to ART initiation were assessed for their ability to neutralize a panel of 34 pseudoviruses from subtypes A, B, C, D, CRFO1_AE and CRF02_AG using a robotic microneutralization assay [17] (**S2 Table**). The panel of viruses was dominated by subtype B, which was largely dictated by a previous study that could predict the neutralization specificity based upon the neutralization pattern, or fingerprint, of well characterized monoclonal antibodies [18]. Therefore, using this viral panel, we were able to determine the neutralization breadth, potency and specificity of neutralizing antibodies following HIV-1 infection. Forty-five samples from 17 donors were excluded from the subsequent analysis due to the detection of non-specific neutralization of MuLV (**S2 Table**), yielding data from a total of 60 placebo and 29 vaccine recipients. Neutralization breadth and potency strongly correlated with each other and increased with time of infection in both groups (**Fig 4A**). Eight (16%) of the infected placebo recipients were able to develop broadly neutralizing antibodies with >70% neutralization breadth three years after HIV-1 diagnosis, whereas none of the individuals who received the RV144 vaccination produced broadly neutralizing antibodies capable of neutralizing 70% of the viral panel (p<0.04, Fischer’s exact test) (**Fig 4A and 4B**). When considering a lower threshold of neuralization breadth, only 3 vaccinated individuals (11%) were able to elicit broadly neutralizing antibodies that could neutralize >50% of the viral panel compared to 15 (31%) placebo recipients (**Fig 4A and 4B**). Lower levels of neutralization breadth were observed as early as year 1 in the vaccine recipients, where none of the vaccine recipients were able to neutralize >50% of the viral panel compared to 13% (5 out of 39) placebo recipients (**Fig 4A and 4B**). We analyzed breadth and potency outcomes using multivariate linear regression models at years 1 and 3 and also evaluated the rate of change. The analysis was conditioned on baseline predictors such as sex, age, risk or coinfection status and viral load. Using this model, neutralization breadth at year 1 was reduced among vaccine recipients compared to placebo recipients (p = 0.041), but not at year 3 (p = 0.261). Neutralization fingerprinting [18] was used to predict the neutralization specificity on samples with >30% neutralization breadth. The membrane proximal external region (MPER) and the CD4 binding site were predicted to be most frequently targeted by neutralizing antibodies measured in both the vaccine and placebo recipients (**Fig 4C and S3 Table**). Overall, there was no difference in the frequency of neutralizing antibody specificities between the vaccine and placebo recipients, with both groups having similar frequency of neutralizing antibodies targeting V2. In summary, these data provide evidence that B cell priming from RV144 vaccination did not accelerate the development of broadly neutralizing antibodies following infection, and instead limited neutralization breadth compared to the placebo recipients.

Since all individuals included in this study were infected with CRF01_AE viruses and the vaccine contained cognate CRF01_AE antigens (A244 gp120 and 92TH023 encoded within ALVAC), we expanded the viral panel to assess neutralization against 14 CRF01_AE viruses (**S4 Table).** We found no evidence that RV144 vaccination augmented the post-infection neutralization response to CRF01_AE viruses. At both time points, breadth and potency did not differ across groups (**S5 Fig**).

In addition, we evaluated the autologous neutralizing antibody responses between vaccine and placebo recipients by generating infectious molecular clones using a CRF01-AE backbone and *env* sequences sampled at the time of HIV-1 diagnosis from 9 vaccine and 14 placebo RV144 recipients. Autologous neutralizing responses were evaluated against 2 to 6 plasma samples, ranging from -500 to 1800 days from the day of diagnosis (**S6 Fig**). Responses were typically weak with no significant difference between ID_50_ values observed for vaccine versus placebo recipients (**S6 Fig**). Since RV144 vaccine efficacy was genotype-specific, we compared responses against viruses with different residues at position 169 and 181. Because vaccine efficacy was strongest toward viruses with K169 or I181X, we expected that, in the vaccine group, neutralization would be stronger against viruses containing the K169 or I181X variants and lower against the vaccine-resistant variants, i.e. those with K169X or I181. Although overall neutralization responses were low, they tended to be higher against K169 than against K169X (p = 0.074) and higher against I181X than against I181 (p = 0.055) (**S6 Fig**), as would be expected from the vaccine efficacy results from the RV144 trial. In addition, stronger binding responses against the CaseA2 protein variant containing K169 were found compared to the wildtype CaseA2 among vaccine recipients, but not when using placebo samples (data not shown).

### B cell priming from RV144 vaccination enhanced Fc effector functions following infection

**Fig 5:**
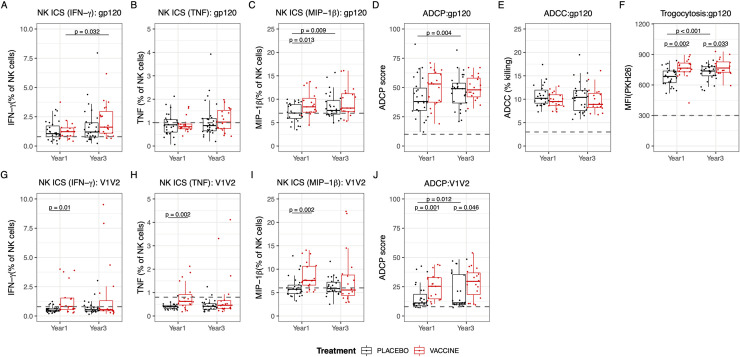
Vaccine recipients demonstrated increased Fc effector functions to V1V2 1 year after HIV-1 diagnosis. Vaccine recipients are represented in red and placebo recipients in black. (A to J) Fc effector function assays to gp120 AE 244 (A-F) or gp70 V1V2 92TH023 (G-J) antigens using year 1 and year 3 samples from vaccine and placebo recipients. Differences in Fc effector function between each group were assessed by NK cell activation (expression of IFNg (A,G), TNF (B,H), or MIP-1b (C,I)), ADCP (D,J), ADCC (E), and trogocytosis (F). The black dashed line represents the positive cut-offs, which were calculated based on two negative controls. Groups were compared using Mann-Whitney U tests, and differences between year 1 and year 3 within the vaccine or placebo groups were calculated using Wilcoxon signed rank test (N = 19 vaccine, 28 placebo). Only significant (<0.05) p values are shown.

Since previous studies have shown that Fc mediated effector functions such as antibody-dependent cellular cytotoxicity (ADCC) and antibody-dependent cellular phagocytosis (ADCP) may have contributed to vaccine efficacy in RV144 [7], we examined the effect of ALVAC/AIDSVAX vaccination on Fc mediated effector function following breakthrough infection (**Fig 5A–5J**). NK cell activation was assessed by intracellular staining (ICS) for IFNγ, TNF, and MIP1β to the cognate vaccine antigens gp120 A244 (AE) and V1V2 92TH023 (AE). Elevated antibody dependent NK cell activation was observed in RV144 vaccine recipients compared to placebo recipients at year 1 post-diagnosis with higher levels of MIP1β when using gp120 as a target antigen as well as higher levels of IFNγ, TNF, and MIP1β when using V1V2 as a target antigen (all p<0.01) (**Fig 5A–5C and 5G–5I**). In addition, B cell priming from RV144 vaccination increased levels of ADCP activity specific to V1V2, while no difference was detected between the vaccine and placebo recipients in ADCP activity to gp120 Env (**Fig 5D and 5J**). The significant differences in NK cell activation and ADCP between the vaccine and placebo recipients waned with time of infection, mainly due to increased functions over time in the placebo recipients reaching levels closer to those of vaccinated individuals (**Fig 5A–5D and 5G–5J**). Interestingly, no differences were detected in ADCC responses to gp120 Env between the groups (**Fig 5E**), but there was a significant difference in trogocytosis to gp120 Env, where the vaccinated individuals exhibited enhanced Fc effector function at both 1- and 3- years post-diagnosis (**Fig 5F**). These data indicate that the Fc mediated effector functions were overall enhanced one year after infection in individuals who were primed by vaccination in RV144 compared to placebo recipients.

### The vaccine profile linked V1V2-specific binding and ADCP responses

**Fig 6:**
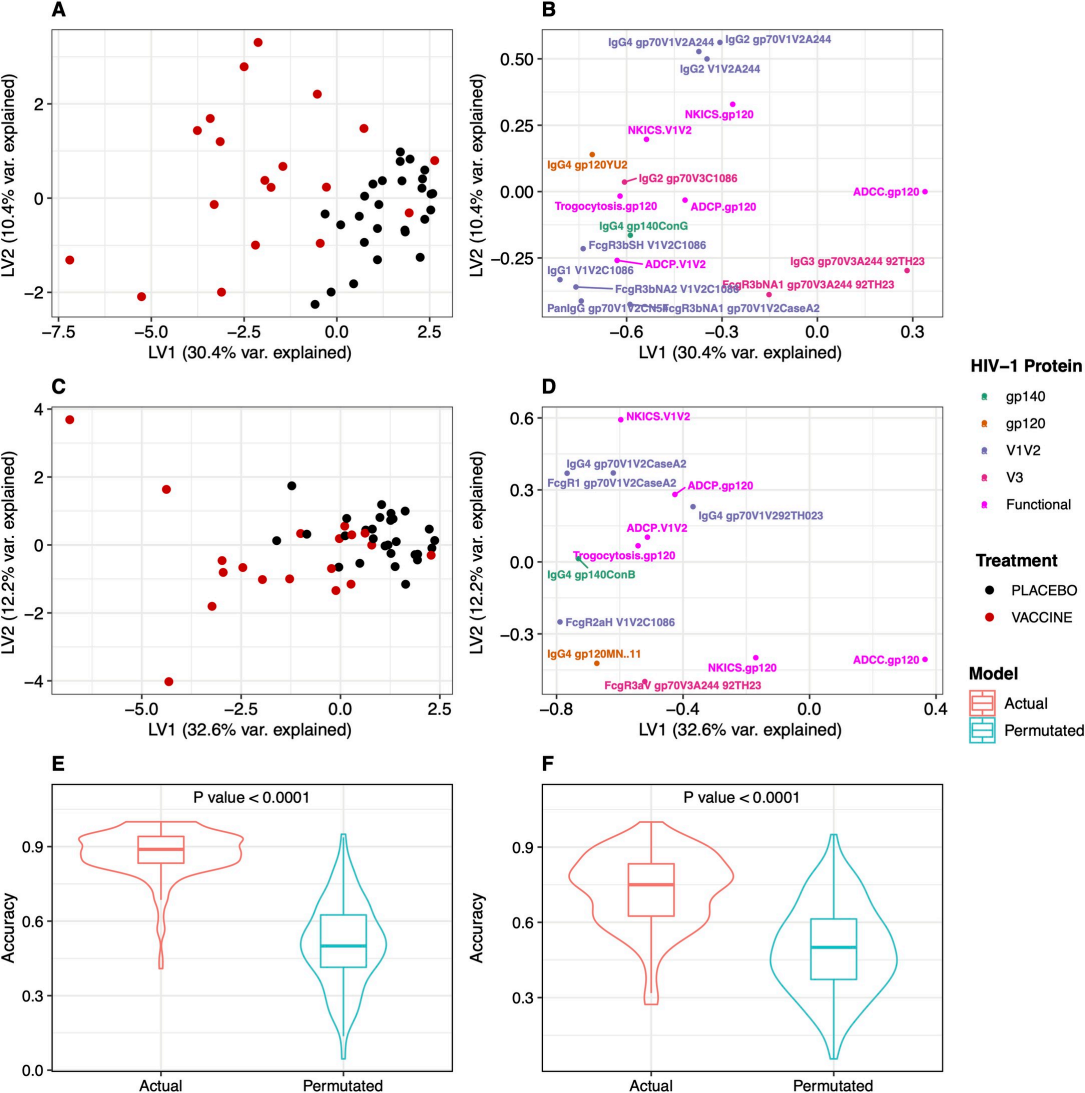
A combination of antibody binding features and Fc effector functions defined a vaccine profile. PLSDA models are shown for year 1 (A, B) and year 3 (C, D) with PLSDA scores on the left and loadings on the right. (D) Cross-validation confirmed that PLSDA models were significantly better than null models generated by permuting treatment groups. (E, F) Performance of PLSDA classification models compared to group permutated models. Cross-validation confirmed that PLSDA models were significantly better than null models generated by permuting treatment groups.

To identify if there was a comprehensive antibody and functional profile associated with vaccine recipients, we used PLSDA classification models combining Ab binding features with the four Fc effector functions assayed: ADCC, ADCP, trogocytosis and Ab-dependent NK cell activation. A distinct combination of immune responses defined the vaccine group and separated ALVAC/AIDSVAX vaccine recipient responses from those of placebo recipients (**Fig 6**). The subset of binding features selected with Random Forest (as described above, **S3 Fig**) (n = 13 at year 1 and n = 7 at year 3) and the 4 Fc effector functions discriminated vaccine participants with a mean balanced accuracy of 90% at year 1 and 70% at year 3 that was significantly different from group-permutated null models (**Fig 6E and 6F**). Differences were largely captured along the first latent variable (LV1), accounting for 31% and 33% of the variance across the treatment groups for year 1 and year 3, respectively. The scores plots confirmed the discriminatory power of the immune variables shown in the loading plots (**Fig 6B and 6D**). The position of the vaccine cluster was associated with different Ab binding and functional responses: IgG4 and V1V2 binding responses, ADCP-V1V2, trogocytosis and NK-ICS showed the strongest associations; (**Fig 6B and 6D**), ADCC responses were not associated with the vaccine cluster.

**Fig 7:**
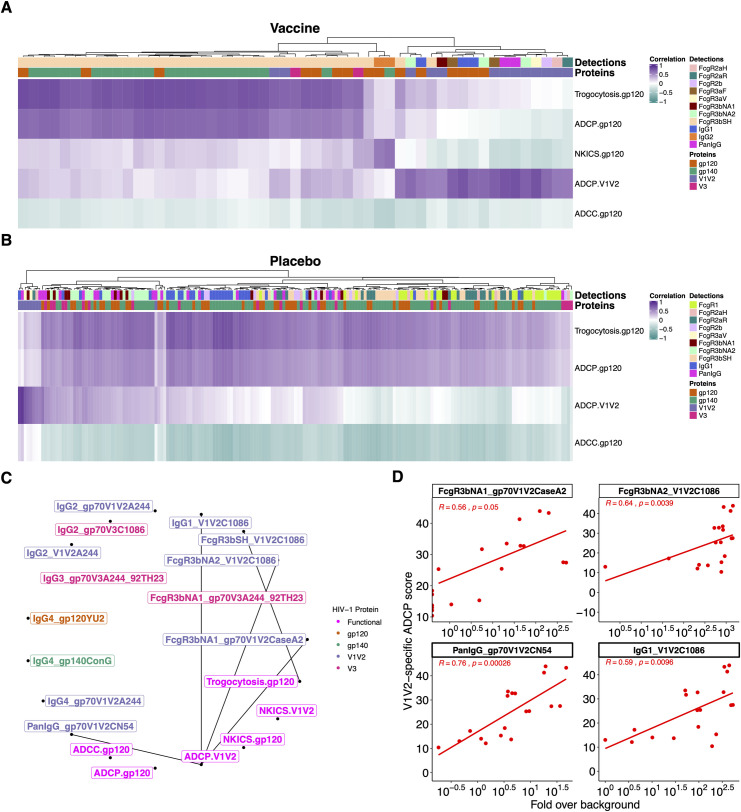
V1V2 responses were associated with V1V2-specific ADCP responses in RV144 vaccinees. Canonical sPLS model highlighted Ab binding features which covaried with the Fc effector functions for vaccine (A) and placebo (B) recipients. Colors show the direction of the associations: positive (dark purple) and inverse (dark cyan). (C) Correlation network highlighting the vaccine RF-selected predictive features that correlated with Fc effector functions. (D) Scatter plots showing significant Spearman correlations between V1V2-specific ADCP and binding responses in vaccine recipients.

After identifying the different features associated with the vaccine group, we evaluated interactions among these Fc binding and Fc effector responses. In the vaccine group, ADCP-V1V2 responses were associated with V1V2 binding responses, and this was observed broadly across FcγR3, FcγR2 and IgG1 (**Fig 7A**). V1V2-specific binding and ADCP responses were the only correlations that remained significant after correcting for multiple comparisons. Placebo recipients showed only a few V1V2 binding features linked to ADCP-V1V2 (**Fig 7B**). Interestingly, the profile associated with V1V2-specific ADCP responses differed from the gp120-specific ADCP profile. As such, gp120-specific ADCP responses were linked to gp140 and gp120 binding responses (**Fig 7A**). The high V1V2-specific ADCP responses in vaccinees were linked to the high V1V2 responses which discriminated vaccine from placebo recipients (**Fig 7C and 7D**). ADCP-V1V2 responses were not significantly correlated with ADCP-gp120 at year 1 (**S7 Fig**).

Next, we determined the Spearman correlations between IgG subclasses and FcγR (Spearman Rho > 0.5, p < 0.05) at each time point to identify whether certain IgG subclasses were specifically associated with Fc effector functions. Only the associations between FcγR and IgG1 or total IgG showed some significant correlations. IgG2, IgG3 or IgG4 responses were typically not significantly associated with FcgR binding (**S7 Fig**). Fc effector functions with significant IgG1 associations were ADCP and trogocytosis (**S7 Fig**), with no other IgG subclass correlated with Fc effector function. In addition, the neutralization breadth seen in the placebo recipients was associated with V3-, gp120- and gp140- specific responses but not V1V2 responses (**S8 Fig**); we note that the virus panel includes Tier 1A viruses (eg, SF162 and 93MW965) which are not indicators of potent neutralizing responses. Overall, vaccine recipients were characterized by a coordinated V1V2-specific ADCP response combined with strong trogocytosis linked with gp120 and gp140 binding antibodies following HIV-1 diagnosis.

### B cell priming from RV144 vaccination did not affect clinical outcomes

The results presented above showed that, following HIV-1 infection, vaccine recipients had distinct immune profiles compared to the placebo recipients. We investigated whether the immune responses measured at peak vaccine induced immunity (two weeks after the last immunization, before infection) correlated with the responses measured after infection. Thirty four vaccinees were included in the RV144 case:controls study and had immune responses measured at peak immunity, a subset of them had experimental results available post-diagnosis at six months (n = 21), one year (n = 18) and three years (n = 24). We found no evidence of a relationship between peak immunity and post-infection measurements for neutralization breadth, ADCC or IgA responses (ADCP and trogocytosis were not measured for the correlates analysis thus precluding comparisons). Beside the small sample size, we note that both the neutralization breadth at peak immunity and the IgA responses post-infection showed very low signal (close to background), possibly explaining the lack of correlation. Binding responses to V1V2 showed some positive correlations especially when IgG3 responses were considered. For example, the pre-infection measurements of IgG3 responses against gp70_V1V2_BCaseA2 were associated with the IgG3 responses against V1V2_A244 measured 6 months post-infection (Spearman Rho = 0.59, p-value = 0). Overall, the small number of participants available for comparison and the time dependence of vaccine-induced immunity (decrease in vaccine-elicited responses over time coupled to the fact that infections occurred at different times after vaccination) complicated the interpretation of the comparison of pre- and post-infection immune responses.

**Fig 8:**
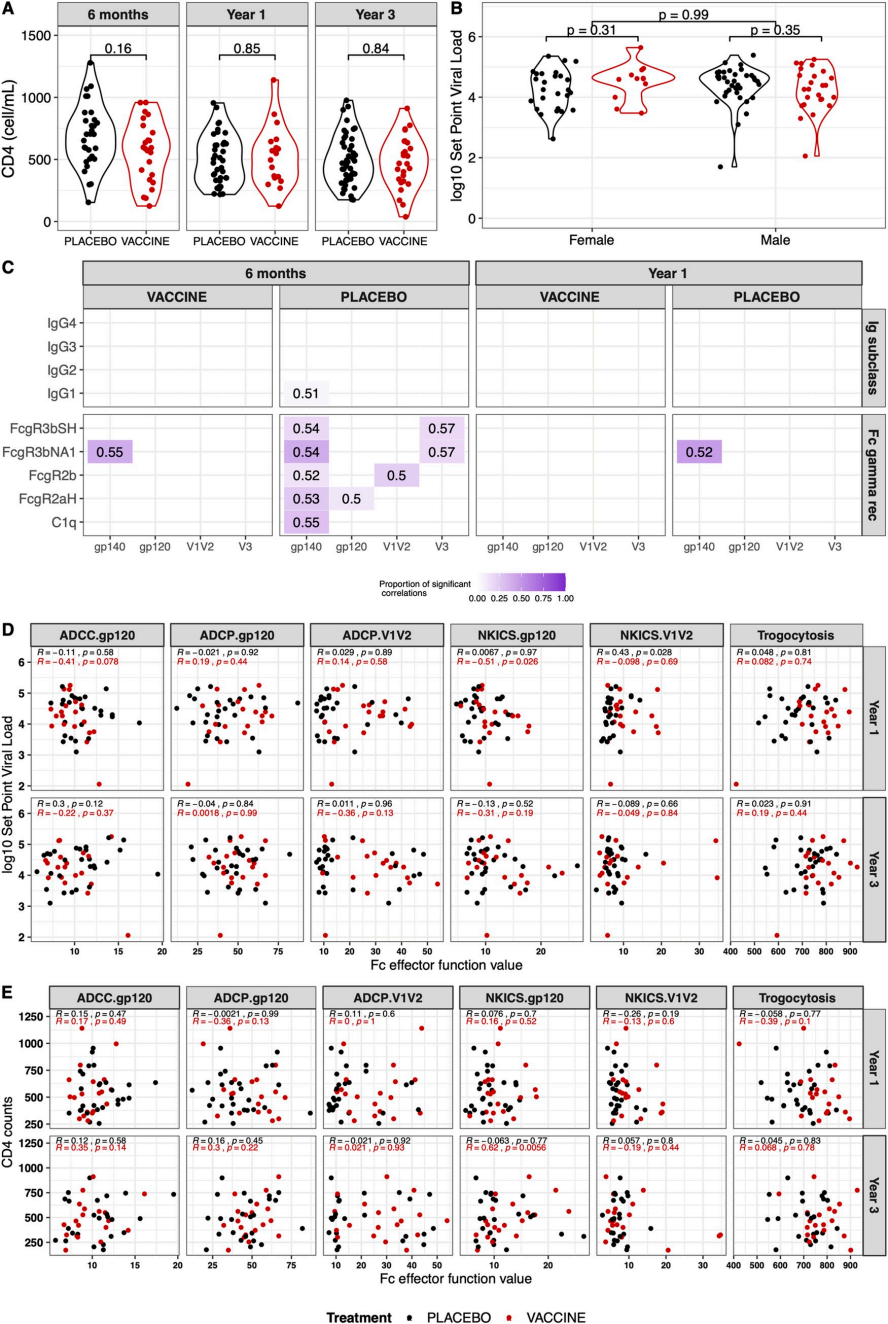
B cell priming from RV144 did not impact markers of disease progression following HIV-1 infection. Vaccine recipients are represented in red and placebo recipients in black. (A) CD4 T cell counts at 6 months (N = 24 vaccine, 31 placebo), year 1 (N = 18 vaccine, 39 placebo) and year 3 (N = 27 vaccine, 49 placebo) and (B) set point viral load (N = 37 vaccine, 63 placebo) showed no significant differences between the vaccine and placebo recipients. (C) Proportion of associations between IgG or FcγR responses and set point viral load that showed significant correlations (Spearman Rho > 0.5, p-value < 0.5). The proportion of significant correlations is shown with a gradient from white to dark purple. The median Rho value of these significant correlations is reported in each cell. (D) No significant correlations between Fc effector functions and set point viral load in vaccine (red) or placebo (black) recipients, except for Env-gp120-specific NKICS assay showed a moderate association in vaccine participants at year 3. (E) No significant correlations between Fc effector functions and CD4 cell counts in vaccine (red) or placebo (black) recipients, except for Env-gp120-specific NKICS in vaccine participants at year 3.

Since the immune profile differed between vaccine and placebo groups, we investigated a potential impact on clinical markers of disease progression. There were no significant set point viral load or CD4 count differences across vaccine and placebo groups (**Fig 8A and 8B**). Only weak associations were seen between IgG responses and set point viral load at 6 months with placebo participants (**Fig 8C**). Moderate correlations between FcγR3b and set point viral load were observed in placebo participants 6 months after diagnosis (**S5 Table**, **Fig 8C**). Fc effector functions were not associated with set point viral load of either vaccine or placebo recipients at year 1 or year 3 (**Fig 8D**). Likewise, most of the Fc effector functions did not correlate with CD4 counts. Only the Env-gp120-specific NKICS assay showed a moderate association in vaccine participants three years after infection (Spearman Rho = 0.62, p = 0.006) (**Fig 8E**). Overall, RV144 vaccine priming of functional antibodies did not impact markers of disease progression for breakthrough participants post HIV-1 infection. Therefore, neither the restriction in neutralization breadth nor enhancement of Fc effector function within the vaccine recipients appeared to affect disease progression.

## Discussion

In this study, B cell priming after RV144 vaccination impacted the specificity, magnitude, dynamics and subclass distribution of the binding antibody response following infection and promoted Fc effector functions while restricting the development of neutralization breadth and potency. The RV144 vaccine strategy focused antibody responses towards V1V2 following infection, suggesting that memory B cells were recalled, yielding increased levels of V1V2 specific binding antibodies. IgG2 and IgG4 responses were boosted in vaccine recipients, while IgG3 responses were lower in the vaccine recipients compared to the placebo recipients. Over time the difference in binding antibody responses between vaccine and placebo groups declined, except for IgG4 responses whereby vaccine recipients still showed significantly higher responses three years after infection. The predominance of IgG4 responses is reminiscent of what was observed in VAX003 participants compared to RV144 participants [7,19,20]. In VAX003, IgG4 responses increased with additional protein immunizations. Interestingly, VAX003 participants received seven protein immunizations (weeks 0, 4, 24, 52, 72, 104 and 144) while RV144 participants received four immunizations at weeks 0, 4, 12, and 24, with proteins included only at weeks 12 and 24. This raises the hypothesis that an additional immunization, like the breakthrough infections observed here, could promote further development of IgG4 responses. Since IgG4 responses are not usually associated with functional effector responses, it will be important to evaluate whether the extra boost included in the HVTN702 Phase III trial triggered IgG4-biased responses and thereby less functional effector responses. The HVTN702 trial, designed as a follow up to RV144, included an additional immunization but failed to show vaccine efficacy (https://www.niaid.nih.gov/news-events/experimental-hiv-vaccine-regimen-ineffective-preventing-hiv).

Unexpectedly, none of the infected vaccinated individuals developed neutralization breadth, while 16% of the placebo recipients were able to achieve neutralization breadth, similar to previous studies in naturally infected cohorts [12,21]. These neutralization differences indicated that the RV144 vaccine recipients were restricted in their development of broadly neutralizing antibodies. Therefore, although there was evidence of B cell priming to V1V2, this priming did not yield V1V2 bnAbs following infection (potent cross-neutralizing responses had also not been observed at peak immunity prior to infection). The lack of V1V2-specific breadth could be linked to differences in binding specificities for RV144 elicited V1V2 Abs when compared to V2-specific bnAbs. While antibodies identified in RV144 vaccinees, such as CH59, targeted a core epitope (HXB2 168–171) that is also recognized by prototypic V2-apex bnAbs, these Abs did not share the characteristics of known V1V2-glycan specific bnAbs such as PG9, CH01, PGT145 or CAP256.09. In addition, we found that the neutralization breadth observed in placebo recipients was not mediated by V1V2 features as associations were found only with Env-V3-, gp120- and gp140- specific responses (**S8 Fig**), and the analysis of the neutralization specificity showed MPER and CD4 binding site targets with no increased specificity towards the V2 apex in the vaccine recipients. With time of infection, breadth and potency increased within RV144 vaccine recipients, and it is possible that these vaccinated individuals will eventually produce broadly neutralizing antibodies. Since studies of neutralization breadth are often concerned with the identification of bnAbs, there is limited data evaluating the early stages of development of bnAbs. Nonetheless, we noted that RV144 placebo participants showed similar neutralization activity as individuals in the RV217 cohort [22] who had been infected for about three years (n = 73) (Shelly Krebs and Samantha Townsley). Although breadth development dynamics could accelerate in vaccinees at later stages of infection, we found no difference in the rate of neutralization development, suggesting that RV144 vaccination primed B cells towards non-neutralizing epitopes that diverted responses away from neutralizing epitopes [23–25]. Priming the B cells towards certain epitopes may have prevented the characteristic Env recognition that has been associated with the elicitation of bNAbs.

An alternative hypothesis is that RV144 vaccine recipients in our study were those who did not mount a strong enough response to prevent infection. The efficacy seen in this trial means that the vaccine selected out intrinsically good V1V2 responders, i.e. vaccine breakthroughs were poor responders to vaccination (they had lower V1V2 binding responses) and may also be individuals with a limited ability to mount bnAbs upon infection. While our comparison of heterologous breadth neutralization (with a multi-subtype panel) indicated that vaccinees were at a deficit compared to placebo participants, we found no evidence of lower responses among vaccinees when we analyzed each participant’s autologous responses, their responses against all viruses from other participants in the cohort and the responses mounted against a set of 14 heterologous but subtype-matched viruses (all individuals in the cohort were infected with CRF01_AE viruses; both the vaccine prime and boost contained CRF01_AE inserts). The vaccinees’s ability to neutralize heterologous but related CRF01_AE viruses contrasts with the restriction in the ability to produce bnAb to more distant viruses comprising distinct subtypes. These findings contradict the hypothesis that vaccinees defined a group with intrinsically less effective HIV-1 immune responses, rather they indicate that the differential humoral responses between the two groups were qualitative and not the consequence of a quantitative impairment among vaccinees. This was confirmed by the stronger V1V2-specific binding and Fc effector antibody functions in the vaccine group than in the placebo group.

Fc effector functions were enhanced in vaccine recipients following HIV-1 infection, including increased NK cell activation targeting V1V2 at year 1, increased ADCP targeting V1V2 at years 1 and 3, and increased trogocytosis to Env at years 1 and 3. Although ADCC was shown to be associated with protection previously, we did not observe a difference in ADCC between vaccine and placebo recipients following infection. In this cohort, the vaccine recipients were characterized by high V1V2-specific Ab binding and ADCP responses. Interestingly, the vaccine priming impact was limited to V1V2-specific ADCP responses whereas gp120-specific ADCP responses were not significantly associated with the vaccine status. In addition, there was no correlation between V1V2- or gp120-specific ADCP responses. This highlights that epitope-based differences can be identified with ADCP assays and warrants the inclusion of different antigens when ADCP is tested. However, although a coordinated V1V2-specific Fc binding and effector profile distinguished the vaccine group from the placebo group, neither this enhanced effector functional profile nor the restriction of neutralization breadth had an effect on HIV-1 set point viral load.

Vaccine mechanisms are typically understood through case-control analyses whereby superior immune responses are identified in vaccine recipients who did not become infected by comparison with the responses elicited in vaccinees who became infected during the trial. Here we highlight a complementary approach which interrogates vaccine-induced immune responses elicited following HIV-1 breakthrough infections. A benefit of such analyses is that we can directly compare vaccine and placebo recipients and evaluate if differences between the groups were due to RV144 vaccination (the fact that the trial was a randomized and double-blinded phase 3 vaccine efficacy trial with no interference allows such evaluation provided that one controls for all baseline participant characteristics that predict HIV-1 infection and the post-infection endpoint) [2]. One limitation of our study was that not all RV144 breakthrough participants were included in our study as some had started anti-retroviral therapy. We were also not able to relate data obtained at peak immunity (prior to infection) to our data obtained post-infection.

In conclusion, we found that vaccination impacted the downstream development of functional antibody responses in individuals who subsequently became infected. While RV144 non-neutralizing antibodies were effective at inducing Fc effector responses, this pathway seemed off-target regarding the development of broadly neutralizing responses, suggesting that vaccine priming dictated the type of immune responses elicited. Immunogenicity studies with Env trimers hint at a parallel phenomenon. Env trimers, which have been regarded as candidate vaccines that could perform better than recombinant proteins (as used in the RV144 trial), have, so far only rarely induced cross-reactive neutralizing responses and these were usually weak [26–33]. These trimers display epitopes such as the V3 loop or the base of the trimer (which do not exist in membrane-bound Env) that are immunodominant but are generally associated with non-neutralizing responses. Owing to their immunodominance, these non-neutralizing antibodies may prevent the elicitation of bnAbs [23–25]. In RV144 participants, the vaccine-induced predominance of B cells with high-affinity for non-neutralizing V2 antibodies suggests that they may out-compete other B cell clones (that could have matured to elicit bnAbs) in the germinal centers and thereby have a negative impact on the development of neutralization breadth in RV144 vaccinees. Hence, our findings illustrate the challenges in the path to a highly efficacious HIV-1 vaccine, as the success of a vaccine may not only derive from the induction of antibodies towards specific epitopes but also depend on the deterrence from other dead-end responses. These results indicate that antigenically imprinting desired responses in the memory B cell repertoire (while preventing other responses) may be critical to develop effective HIV-1 vaccines.

## Materials and methods

### Ethics statement

All participants signed informed consent and participated in MHRP protocols approved by Thai and Walter Reed Army Institute of Research (WRAIR) Institute Review Boards.

### Study design

This study included samples obtained from vaccine and placebo participants who became HIV-1 infected in the RV144 Phase III vaccine clinical trial (NCT00337181). Samples were obtained prior to initiation of antiretrovirals. For antibody profiling: 188 samples from 6 months (29–266 days; 24 vaccine, 31 placebo), year 1 (range 295–454 days; 18 vaccine, 39 placebo) and year 3 (805–1188 days; 27 vaccine, 49 placebo). For neutralization responses: 37 vaccine and 63 placebo.

### Cell lines

Human embryonic kidney (HEK)-derived 293T and HEK293S N-acetylglucosaminyltransferase I-negative (GnTI-) cells were obtained from the American Type Culture Collection (ATCC), and HeLa-derived TZM-bl reporter cells were acquired through the NIH AIDS Reagent Program (ARP). HEK293T, HEK293S GnTI-, and TZM-bl cells were maintained in complete Dulbecco’s Modified Eagle Medium (herein referred to as cDMEM) containing high glucose Dulbecco’s Modified Eagle Medium (DMEM, Thermo Fisher), 1X Penicillin-Streptomycin (Pen Strep, Thermo Fisher) and 10% fetal bovine serum (FBS, Gemini Bio Products) at 37°C/5% CO_2_. Expi293F cells (Thermo Fisher), were maintained in Expi293 Expression Medium (Thermo Fisher), at 37°C/10% CO_2_ with shaking at 120 RPM. The following reagents were obtained through the NIH AIDS Reagent Program, Division of AIDS, NIAID, NIH: pSG3Δenv from Dr. John C. Kappes and Dr. Xiaoyun Wu [34,35]; pSV-A-MLV-env from Dr. Nathaniel Landau and Dr. Dan Littman [36]; TZM-bl cells from Dr. John C. Kappes, Dr. Xiaoyun Wu and Tranzyme Inc [37].

### Viral load and CD4 T cell counts

Testing of viral load and CD4 T cell counts was performed every six months. Set point viral load was calculated using all samples obtained between 28 and 365 days after diagnosis.

### Protein production

Uncleaved founder gp140 proteins incorporated the native leader peptide, an R to S mutations in the gp120/gp41 cleavage site, and full MPER sequences followed by a short GGGS linker sequence and a C-terminal AviTag. Sequences, codon-optimized for expression in human cells, were synthesized (Genscript) and cloned into a custom pcDNA3.4 expression vector (ThermoFisher) using sequence- and ligation-independent cloning (SLIC) methods. Proteins were expressed by transient transfections in Expi293 cells (ThermoFisher) according to the manufacturer’s instructions. Gp140 proteins were purified from clarified cell culture supernatants 4 days post-transfection using *Galanthus nivalis* lectin (GNL) resin (Vector Labs) affinity chromatography. When needed, proteins were further flowed through a Q-sepharose (GE Healthcare) column to remove host cell protein contaminants. All gp140s were purified to 90% purity or higher, as assessed by SDS-PAGE and Coomassie staining in reducing and non-reducing conditions. An additional post-purification control step included binding to CD4-Ig, as measured by BLI, to insure proper gp140 folding.

### Antibody profiling

Multiplex antibody binding and characterization was performed as previously described [16,38, 39] with minor modifications. Briefly, heat-inactivated human plasma samples were loaded into 384-well assay plates by use of a Biomek NXP automated liquid handler (Beckman Coulter, Atlanta GA). All samples were tested at two dilutions (1:100 and 1:1000) in duplicate wells. Pooled HIV+ plasma, pooled normal human plasma, HIV-IG and normal human IgG and no-sample control wells were included on each assay plate.A panel of 56 antigens, including a diverse array of HIV-1 antigens and positive and negative controls was used and supplemented with antigens obtained from Immune Tech (New York, NY), the Duke University Protein Production Facility (Durham, NC) and the NIH AIDS Reagent Program (Bethesda, MD) (**S1 Table**). Each antigen was covalently coupled to uniquely coded carboxylated magnetic microspheres (Luminex Corp., Austin TX) per manufacturer’s protocol and as described [16,38]. Microspheres are activated by incubation in buffer containing 1-Ethyl-3[3-dimethylaminopropyl]carbodiimide hydrochloride and N-hydroxysulfosuccinimide for 20 min. The cocktail of 56 antigen-coated microspheres were added to the plate in a final volume of 50 μL/well. Following a 2 hr incubation with vigorous shaking, microspheres were washed using a magnetic 384-well automated plate washer (Bio-Tek, Winooski VT) to remove unbound sample. Microspheres are then resuspended with 40 μL PE-labeled detection reagent, vortexed for 1 min with a microplate vortex at 3000 rpm (VWR, Radnor PA), sonicated for 1 min in an ultrasonic bath (Branson Ultrasonics, Danbury CT) then incubated with vigorous shaking for 1 hr. After a final wash to remove unbound detection reagent, microspheres are resuspended in 40 μL sheath fluid (Luminex) as above. Data was collected on a Bio-Plex 3D Suspension Array system (Bio-Rad, Hercules CA) running xPONENT v.4.2 (Luminex Corp).

Ig subclass and isotype specificity were determined by detection with R-phycoerthrin (PE)-conjugated mouse anti-human IgG, IgG1-4 and IgA (Southern Biotech, Birmingham AL) at 0.65μg/mL. Human complement component C1q (Sigma-Aldrich) was biotinylated using the EZ-Link NHS-PEG4-Biotin kit (ThermoFisher Scientific). Following labeling, excess biotin was removed by passage over a Zeba desalting column (ThermoFisher Scientific). Biotinylated C1q was labeled and tetramerized with Phycolink Streptavidin-PE (Prozyme, Hayward CA) at a 1:4 molar ratio for 30 min, quenched with excess free biotin and stored at 4°C for up to 48 hours before use.

### Production of pseudoviruses

Pseudoviruses for use in TZM-bl neutralization assays were produced in 293T cells by cotransfection of a pSG3ΔEnv backbone plasmid and a full HIV-1 Env gp160-encoding plasmid [40]. Env sequences were selected with functional open reading frames from the HIV-1 genome sequences that had been derived from plasma samples collected at HIV-1 diagnosis. These env genes were cloned into a replication-competent HIV-1 vector derived from a CRF01_AE genome isolated from a Thai participant enrolled in the RV217 cohort.

Briefly, 2X10^6^ cells in 20ml cDMEM were seeded in T75 flasks the day prior to cotransfection. For transfection, 40μl of FuGene 6 reagent (Promega) was diluted into 800μl of room-temperature Opti-MEM I reduced serum medium (Thermo Fisher), followed by addition of 10μg of pSG3ΔEnv backbone plasmid. 3.3μg of HIV-1 Env plasmid was then added to the mixture, mixed, and incubated for 30 minutes at room temperature. Transfection mixture was then added to media of previously seeded 293T cells in the T75 flask and then distributed evenly on cells. The following day, media was replaced with 20ml fresh cDMEM. Virus was harvested the following day by filtering cell supernatants with 0.45μm Steriflip units (EMD Millipore) and aliquoted. Pseudoviruses capable of a single round of infection were produced by co-transfection of HEK-293T cells with plasmids expressing *env* and pSG3ΔEnv backbone. Pseudovirus stocks were titrated by luciferase reporter gene expression in TZM-bl cells as previously described [40] by targeting 100,000 relative light units (RLU) for each pseudovirus after a 48-hour incubation period.

### Neutralization

Samples from infected RV144 individuals were evaluated for NAbs at 1–3 years post-diagnosis prior to the initiation of ART. Using a high-throughput robotic microneutralization assay, samples were analyzed against a panel of 35 viruses from subtypes A, B, C, D, CRF_01 AE, and CRF_02 AG. Included within this panel were 21 pseudoviruses where the pattern of neutralization can predict NAb specificity. Neutralization breadth, potency, and specificity of the responses observed after infection were compared between the vaccine and placebo recipients.

Neutralizing activity was assessed with a recombinant virus assay that included a reference panel of full-length *env* pseudo-typed viruses previously selected to assess neutralization breadth and to map epitope specificity of plasma antibodies by the breadth of their neutralizing antibody response {Georgiev, 2013 #45}. HIV-1 *env* DNA was obtained from the NIH ARP, James Binley (Torrey Pines Institute), David Montefiori (Duke University Medical Center) John Mascola (VRC, NIH/NIAID), and Dana Gabuzda (Dana Farber Research Center). 192 samples from 72 individuals who were HIV-1 infected were evaluated for bNAbs at 1 and 3 years post diagnosis. Using a microneutralization assay, samples were analyzed against a panel of 35 viruses from multiple subtypes. Neutralization was measured by reduction of luciferase gene expression as previously described (Montefiori, 2005). Briefly, indicator virus was incubated with serial 4-fold dilutions of plasma samples in duplicate before being added to TZM-bl cells. After a 48-hour incubation, luciferase activities were measured using Britelite Plus Reporter Gene Assay System (Perkin Elmer) substrate solution. Neutralization activity was expressed as the reciprocal plasma dilution that resulted in 50% reduction (ID_50_) of RLU. Luminescence was read using the Molecular Devices Paradigm LUM384 module and data was uploaded into MHRP Labkey analysis server to obtain ID_50_ values. Positive neutralization was defined as 50% inhibition of infection of an HIV-1 strain at ≥1:40 plasma dilution, 2-fold higher than the limit of detection for the assay, and less than 50% inhibition of infection of murine leukemia virus (MuLV) was detected. Breadth was calculated as the percent of the 35-virus panel neutralized by ≥1:40 plasma dilution. Potency was calculated by the geometric mean titer (GMT) of the ID50 of all 35 viruses within the panel.

### Neutralization fingerprinting analysis

Delineation of neutralization specificity was performed as previously described [18] using 30 pseudoviruses out of the 35 viral panel. For a given sample, the approach compares a polyclonal neutralization pattern of a set of diverse viral strains to the neutralization patterns (or fingerprints) of a reference set of broadly neutralizing mAb specificities, to obtain an estimate of the contribution of each of the reference specificities to polyclonal neutralization [18,41]. We applied computational quality control metrics for filtering out plasma samples for which the predictions were deemed unlikely to be accurate [41]. Through this process, the initial set of samples was reduced to 80 samples, which were used for the analysis of Ab specificity frequency. Samples were predicted to have between 1 and 3 specificities from the reference set, and the overall frequency of observing the different reference specificities or pairs of reference specificities were analyzed.

### ADCP

ADCP was measured as previously described [42]. Briefly, gp120 or gp70 V1V2 (Case A2) were biotinylated at a biotin (Thermo Scientific) to antigen ratio of 50:1 following manufacturer’s instruction and incubated with yellow-green strepavidin-fluorescent beads (Molecular Probes) 2h at 37°C. A 100-fold dilution of beads–gp120 (10μl) was incubated 2h at 37°C with 100μl of diluted plasma samples before addition of THP-1 cells (20,000 cells per well; Millipore Sigma, Burlington, MA, USA). After 19h incubation at 37°C, the cells were fixed with 2% formaldehyde solution (Tousimis, Rockvile MD USA) and fluorescence was evaluated on a BD LSRII instrument. The phagocytic score was calculated by multiplying the percentage of bead-positive cells by the geometric mean fluorescence intensity (MFI) of the bead-positive cells and dividing by 10^4^.

### NK ICS

96-well plates were coated with 1.5μg/ml of gp120 or gp70 V1V2 overnight at 4°C and washed with PBS. Diluted plasma samples were added, incubated at 25°C for 2 hours, and washed with PBS. Cryopreserved PBMCs from one healthy human donor were added at 1 million cells/well and incubated at 37°C for 6 hours in presence of Brefeldin A and Monensin (BD Bioscience).

Cells were surface stained with anti-CD3 AF700 (Clone UCHT1, BD Pharmingen), anti-CD56 PE-Cy7 (Clone B159, BD Biosciences), anti-CD16 APC-Cy7 (Clone 3G8 BD Biosciences), anti-CD19 BV510 (Clone HIB19, BD Biosciences), anti-CD14 BV510 (Clone M5E2, Biolegend) and with Live/dead Fixable Aqua Cell Stain (Thermo Fisher Scientific). Cells were fixed and permeabilized (Cell Fixation & Permeabilization Kit, ThermoFisher) and stained intracellularly for TNF (Clone Mab11, Biolegend), IFNγ (Clone B27, BD Biosciences), and MIP-1β (Clone D21-1351, BD Biosciences). Data were acquired on a BD LSRII instrument and analyzed using FlowJo Version 9.9.6 software. NK cells were gated as follows: singlets (FSC-H v. FSC-A); Aqua-negative; low side scatter; triple negative for CD3, CD19, and CD14; and either CD56+CD16-, CD56+CD16+, or CD56-CD16+ (**S9 Fig**).

### Trogocytosis and ADCC

Trogocytosis and ADCC were measured using our previously published assay [43]). Briefly, CEM.NKR.CCR5 cells (NIH AIDS Reagent Program, Division of AIDS, NIAD, NIH courtesy of Dr. Alexandra Trkola [44]) were washed with PBS and stained with PKH26 (Sigma-Aldrich, St-Louis, MO, USA) at 2 μM in Diluant C at room temperature for 5 minutes. Cells were then washed with RPMI 10% FBS (R10), resuspended in R10, and incubated with gp120 (Case A2) for 1 hour at room temperature. Cells were then washed twice with R10 and incubated with diluted plasma samples for 1 hour at room temperature. Effector cells (PBMC form one healthy subject) were then added in R10 at an effector to target cell ratio of 50:1 and incubated for 5 hours at 37°C. After the incubation, cells were washed, stained with Live/dead aqua fixable cell stain (Life Technologies, Eugene, OR, USA) for 15 minutes at room temperature, washed, and fixed with 2% Formaldehyde (Tousimis, Rockville, MD) for 15 minutes at room temperature. Data were acquired on a BD LSRII instrument (BD Biosciences) and analyzed using FlowJo Version 9.9.6 software (TreeStar, Ashland, OR, USA).

### Statistical analysis

Statistical analysis and visualization were performed using R statistical computing environment (with R packages ggplot2, gplot, rpart and mixOmics) [45]. Statistical significances were computed using the Mann-Whitney U test and Spearman correlation coefficients were calculated with two-tailed p values. False Discovery Rate (FDR) was applied to correct for multiple testing to control type-I error (p < 0.05 for significance unless otherwise stated). The ComplexHeatmap library in the R environment was used for hierarchical clustering which grouped the immune measurements (detections and antigen features) by their similarities. Data were standardized before clustering by centering around zero and scaling to the standard deviation of one for each feature. For all neutralization assays, data were fitted to a 5-parameter asymmetric nonlinear regression model to obtain the IC50, or concentration of mAb/dilution of plasma needed to obtain 50% neutralization against a given pseudovirus. For neutralization assays in which a fold-change in IC50 imparted by a particular virus mutant, virus treatment, or binding stoichiometry (e.g., IgG vs. Fab) was reported, the IC50 obtained for one virus/assay condition was divided by the IC50 obtained for the other virus/assay condition, as indicated in the figure legends and *y*-axes of data graphs. All neutralization assays were repeated at least 2 times, and data shown are from representative experiments. Analysis was performed within R 3.6.0 through R Studio (1.1.453, R Consortium, Boston, MA). Figures were created using GraphPad Prism 7.0 (GraphPad Software, La Jolla, CA, USA) and R 3.6.0.

### Correlation filtering

Collinear variables were removed using polyserial correlation to produce stable Random Forest and PLSDA discriminators. Polyserial correlation was applied to rank features according to their correlation with a treatment group while Spearman correlation was used to identify correlated features. A subset of high-quality non-redundant features was identified by removing correlated features. Features were considered in decreasing order of polyserial correlation coefficient, removing subsequent features that were highly correlated (p > 0.8) to those already selected.

### Random Forest and Classification and Regression Trees (CART)

A combined modeling approach integrating Random Forest and CART was adopted from [46]. The modeling method was performed with variables selected after the correlation filter. Random Forest processes were adapted as an additional variable selection procedure to improve the stability of the model. Random Forest selection was carried out independently for each HIV-1 antigen (gp120, gp140, V1V2 and V3). A Random Forest importance score metric was used to rank features according to their importance in classifying groups. A Random Forest algorithm (‘RandomForest’ function in R) was evaluated by drawing a bootstrap sample from the data (75% of the data) and variable importance scores were generated for each feature. The process was repeated to generate 200 random bootstrap samples to improve stability. Features were used into CART models if they achieved an average importance score of 70% or above for each antigen. An exploratory tree was then grown with the relevant features selected through random forest using the ‘rpart’ function in R package. The rpart algorithm splits the data recursively until a predefined termination criterion is met. The splitting at each iteration is based on a variable that results in the maximum reduction in the heterogeneity of a selected variable. The Gini Index, which measures how often a randomly selected variable incorrectly labels the designed groups, is used as a measure of heterogeneity. To evaluate model performance, AUC curves were created (i) using cross-validation on training data at each time point and (ii) using testing data from a different time points to the data used to train the model.

### Partial Least Square Discriminant Analysis (PLSDA)

PLSDA models were generated with Random Forest-selected non-redundant features to assess their ability to classify the treatment groups. The function ‘plsda’ in the mixOmics R package was used to generate each model at each of the time points by setting the number of components to 10 with all other parameters kept at default. The performance of each of the PLSDA models was assessed using permutation tests. We permuted the treatment groups to create a null model and recalculate the model using the permutated data as described above. The data was permuted 200 times and 10-fold cross validation was used to evaluate the goodness of fit of the model. A distribution of balanced of accuracy metrics obtained with the non-permutated/true groups was compared with that obtained with the permutated groups (null hypothesis) classification. The assumption was that the classification model created with the permutated data should fail to predict group classes.

### Partial Least Square (PLS) analysis

PLS models were generated to link Ab biophysical binding features to Fc effector functional features. Both PLS models with all the features and the sparse variation (sPLS) were used. sPLS combines both integration and variable selection in a one-step strategy to maximize the covariance between two data sets and identify latent variables. Univariate Spearman correlations were first generated for each pair of biophysical and Fc effector functional features, selecting features with a significant correlation coefficient (FDR < 0.05) to be included in the PLS model. The sPLS process was performed using the `sPLS’ function from the mixOmics package in the R environment. We tuned the number of components (ncomp) using an ‘elbow’ method, selecting the ncomp where we observed a diminishing change in the variance captured as we increased ncomp. The number of variables to keep in each component was tuned using nfold cross validation repeated 100 times, selecting the number of variables resulting in the lowest MSE value for each component. Associations/links were visualized using heatmaps generated with the ‘cim’ function in the mixOmics package. sPLS models were generated separately for each treatment group in order to discern distinct humoral relationships in the vaccine group.

### Multinomial logistic models

LASSO-regularized multinomial regressions were adapted from [47]. Logistic classifiers were trained with the features selected through correlation filtering as mentioned above. The classifier models were trained within the R package using the `glmnet’ function with lambda selected through a 10-fold cross-validation. A final model used for visualization was trained using the model with the lowest cross-validation classification error. To assess the predictability of the classifier, 200 repetitions were performed evaluating the balanced accuracy of the 10-fold cross-validations with training and testing sets randomly selected internally by the ‘glmnet’ function. Note that low numbers in some groups may not guarantee that each fold had at least a sample available and that the uneven distribution between groups may not guarantee equal numbers of samples in each fold. Robustness was tested for the classifier using permutation testing. Group labels were permutated by randomly shuffling the treatment arm. A logistic classifier was then evaluated for the data with permutated groups through the same process as with the original data. This process was repeated 200 times, each time retrieving the balanced accuracy of the 10-fold cross-validations. A comparison of the distribution of balanced accuracy between the actual and permutated groups was analyzed in terms of effect size and the tail probability of the mean balanced accuracy of each distribution.

## Supporting information

S1 Table HIV-1 antigens used in profiling antibody responses. 52 HIV-1 antigens were used to profile binding antibody responses using multiplex Luminex assays, including 9 gp120s, 24 gp140s, 9 V1V2 and 5 V3 antigens. Stars designate antigens used in the RV144 correlates study.
(XLSX)

S2 Table Neutralization titers of 180 samples from 106 individuals following HIV-1 infection. Samples (listed vertically) were assessed at 1- and 3- years post-diagnosis. Breadth indicates the percentage of viruses neutralized from the panel of 34 diverse viruses (listed at top horizontally) with an ID40>40. Potency indicates the geometric mean titer (ID50) of all viruses. Non-specific neutralization as mediated by MuLV. Plasma that neutralized MuLV ID50 >20 was excluded from analysis (N = 45 samples). The table was sorted by MuLV neutralization, Treatment Group, and year from diagnosis.
(XLSX)

S3 Table Delineation of neutralizing antibody specificity from vaccine and placebo recipients at 1 and 3 years post-diagnosis. Thirty viruses were used to predict the specificity of the neutralizing antibodies with a neutralization breadth >30%. Epitope specificities were designated for each algorithm if the value was >0.3 for the respective monoclonal antibodies. If no specificity gave a value >0.3, the sample was designated as ‘unspecified’. The table was sorted by treatment group and time from diagnosis.
(XLSX)

S4 Table Neutralization titers of CRF01_AE viruses following HIV-1 infection. Samples were assessed at 1- and 3- years post-diagnosis. aBreadth = % of viruses neutralized from the panel of 14 subtype AE viruses (top) with an ID40>40. bPotency = Geometric mean titer (ID50) of all viruses. Non-specific neutralization as mediated by MuLV. Plasma that neutralized MuLV ID50 >20 was excluded from analysis (N = 45 samples). The table was sorted by MuLV neutralization, Treatment Group, and Year from Diagnosis.
(XLSX)

S5 Table Significant correlations between Fc binding features measured at 6 months and set point viral load in placebo participants. Spearman correlations were evaluated between 816 Fc binding responses and set point viral load. Only correlations with Spearman Rho ≥ 0.5 and significant p-values after false discovery rate corrections with 0.5 are reported.
(XLSX)

S1 Fig Dynamics of IgG subclasses between RV144 vaccine and placebo recipients following infection. The proportion represented by each subclass is represented at 6 months, 1 and 3 years post-diagnosis. Significant changes in the subclass ratio between time points are demonstrated (*** for p <0.001, ** for p < 0.01 and * for p < 0.5) using Wilcoxon signed-rank test for both vaccine (V) and placebo (P) participants.
(TIFF)

S2 Fig Total IgG and IgA binding antibody responses in vaccine (red) and placebo (black) recipients. (A) Total IgG and (B) IgA binding to HIV-1 Env antigens at 6 months (N = 24 vaccine, 31 placebo), year 1 (N = 18 vaccine, 39 placebo) and year 3 (N = 27 vaccine, 49 placebo) post-HIV-1 diagnosis. Composite scores are the geometric mean of fold over background per antigen consisting of 9 gp120s, 23 gp140s, 6 V1V2 gp70, and 4 V3 gp70 antigens. p values were calculated by Mann-Whitney test and adjusted by False Discovery Rate (FDR) for multiple comparisons. Only significant (p<0.05) p values are shown.
(TIFF)

S3 Fig Binding features ranked by the average importance score of each variable at (A) 6 months, (B) year 1 and (C) year 3. Binding features are considered separately for each time point and HIV-1 antigen. Variables with a score above 70% (dashed line), corresponding to a 30% mean decrease in accuracy, are considered relevant.
(TIFF)

S4 Fig Fc biophysical features associated with treatment arms at 6 months, 1 and 3 years post-diagnosis identified with a multinomial logistic model. IgG4-gp120 responses and FcγR responses against V1V2 were robustly selected by LASSO to classify treatment arms. Left panel: coefficient weights for the final logistic regression model. Middle panel: percentage of each feature across CV folds and replicates for the features selected in the final logistic regression model. Right panel, top plot: visualization of the top two logistic regression coefficients by magnitude; vaccine recipients are represented in red and placebo recipients in black. Right panel, bottom plot: performance of the logistic regression classification from repeated cross-validation using actual versus permutated data with a one-sided P-value and for 200 independent repetitions; the effect size measured with Cliff’s delta shown in the figure.
(TIFF)

S5 Fig Neutralization of CFR01_AE pseudoviruses of vaccine and placebo recipients at year 1 and year 3 post-HIV-1 diagnosis. (A) Neutralization breadth (top) and potency (bottom) of vaccine (in red) and placebo (in black) recipients using the panel of 14 AE pseudoviruses compared in aggregate using Mann-Whitney t tests. (B) Spearman correlations of neutralization breadth and potency (geometric mean titer (GMT)) of placebo (top) and vaccine (bottom) recipients at year 1 (N = 12 vaccine, 25 placebo), and 3 (N = 26 vaccine, 43 placebo), post-diagnosis. Neutralization breadth is the percentage of viruses neutralized out of a panel of 14 CRF01_AE pseudoviruses.
(TIFF)

S6 Fig Autologous neutralizing antibody responses in RV144 vaccine and placebo recipients and responses across participants within treatments groups. (A-B) Longitudinal autologous neutralization in vaccine (N = 9, red) and placebo (N = 14, black) recipients following HIV-1 diagnosis. (C-D) Neutralization responses against envelopes with or without residues associated with vaccine efficacy in RV144 (responses were measured in a matrix format against viruses from the 23 participants with available pseudoviruses). The HIV-1 Env variants associated with vaccine efficacy were K169 and I181X.
(TIFF)

S7 Fig Associations between IgG responses and Fc gamma receptor features or Fc effector functions. Panels A and B show the proportion of significant correlations (Spearman Rho > 0.5, p < 0.5) between IgG responses and either FcγR responses (A) or Fc effector function (B). The color represents the proportion of significant associations whereby higher proportions are marked in dark purple. The median Rho value of these significant correlations across all FcγR features is reported in each cell. (C) Significant Spearman correlations between Fc effector functions within each treatment groups. Blue: positive correlation; Cyan: negative correlation. Stronger colors represent significant correlations after correction for multiple testing.
(TIF)

S8 Fig Neutralization breadth and potency associated with V3-, gp120- and gp140-specific features. (A) Ig features associated with neutralization breadth or potency with Spearman Rho ≥ 0.5 (p-value ≤ 0.05). (B) Canonical sPLS model highlighted Ab binding features which covaried with the neutralization data in the placebo group at year 3 post-diagnosis.
(TIFF)

S9 Fig Gating strategy for the Fc-mediated antibody effector functions. The gating strategy and representative results for one positive and negative control are shown for ADCP, trogocytosis, ADCC, and NK cell activation.
(TIFF)
