# Correction: RV144 HIV-1 vaccination impacts post-infection antibody responses

**DOI:** 10.1371/journal.ppat.1009386

**Published:** 2021-03-02

**Authors:** Thembi Mdluli, Ningbo Jian, Bonnie Slike, Dominic Paquin-Proulx, Gina Donofrio, Aljawharah Alrubayyi, Syna Gift, Rebecca Grande, Mary Bryson, Anna Lee, Vincent Dussupt, Letzibeth Mendez-Rivera, Eric Sanders-Buell, Agnès-Laurence Chenine, Ursula Tran, Yifan Li, Eric Brown, Paul T. Edlefsen, Robert O’Connell, Peter Gilbert, Sorachai Nitayaphan, Punnee Pitisuttihum, Supachai Rerks-Ngarm, Merlin L. Robb, Robert Gramzinski, Galit Alter, Sodsai Tovanabutra, Ivelin S. Georgiev, Margaret E. Ackerman, Victoria R. Polonis, Sandhya Vasan, Nelson L. Michael, Jerome H. Kim, Michael A. Eller, Shelly J. Krebs, Morgane Rolland

The twelfth author’s name is spelled incorrectly. The correct name is: Letzibeth Mendez-Rivera.
