# Supplementary figures and images for: RV144 HIV-1 vaccination impacts post-infection antibody responses
Source: PLoS Pathog. 2020 Dec 8;16(12):e1009101. doi: 10.1371/journal.ppat.1009101 (PMC7748270; doi:10.1371/journal.ppat.1009101)

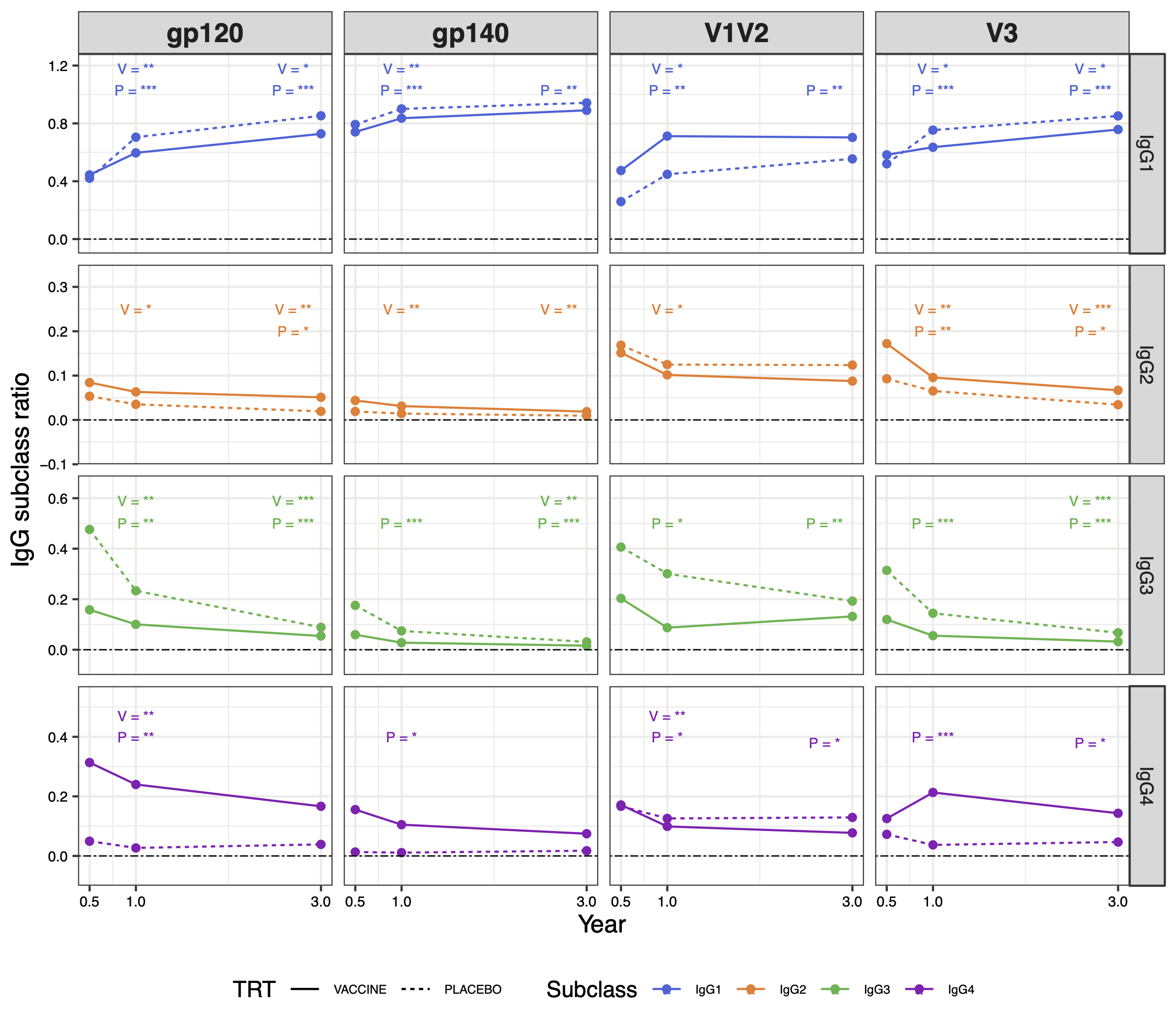

Supplement: S1 Fig — The proportion represented by each subclass is represented at 6 months, 1 and 3 years post-diagnosis. Significant changes in the subclass ratio between time points are demonstrated (*** for p <0.001, ** for p < 0.01 and * for p < 0.5) using Wilcoxon signed-rank test for both vaccine (V) and placebo (P) participants. (TIFF) [file ppat.1009101.s006.tiff]

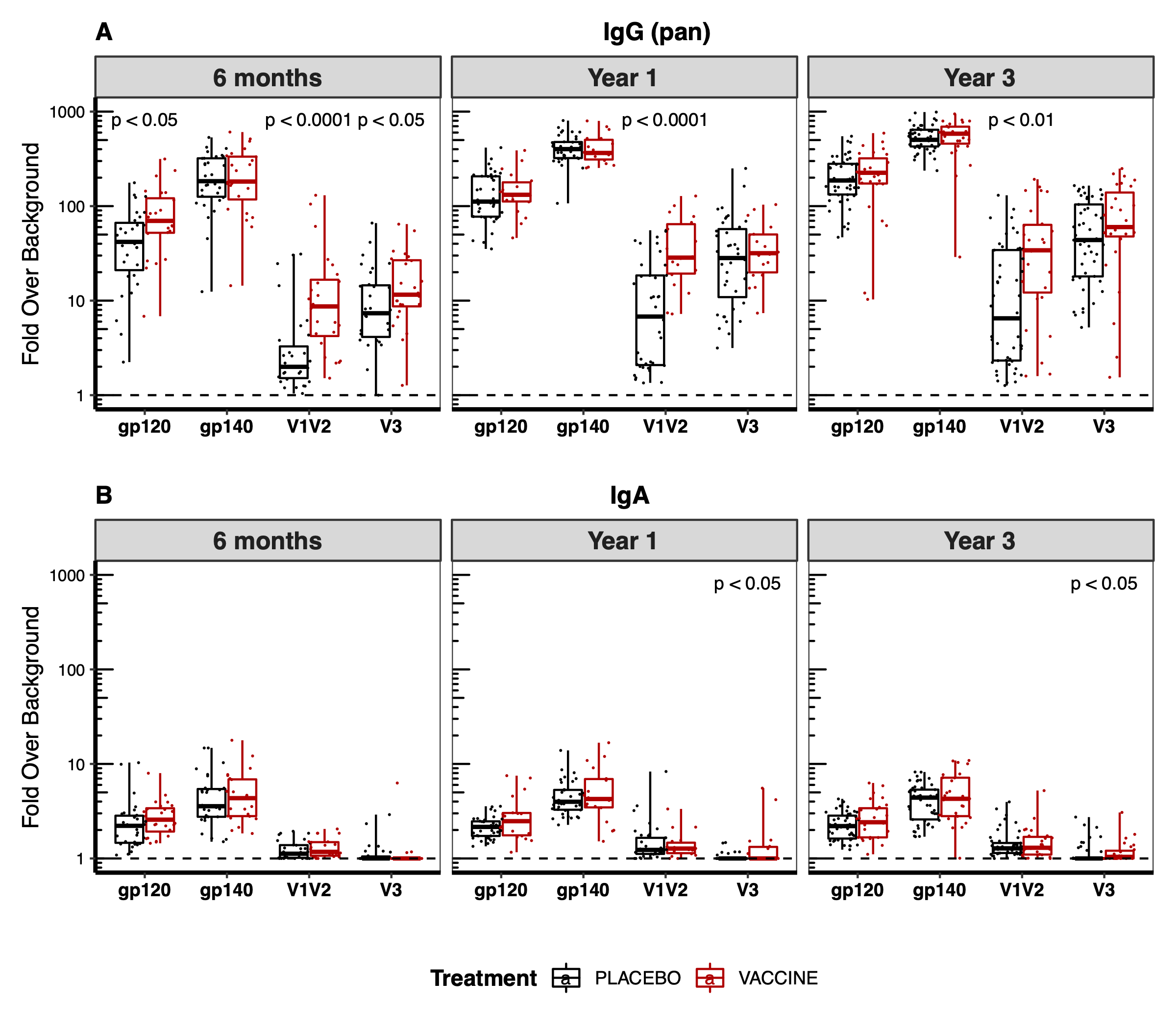

Supplement: S2 Fig — (A) Total IgG and (B) IgA binding to HIV-1 Env antigens at 6 months (N = 24 vaccine, 31 placebo), year 1 (N = 18 vaccine, 39 placebo) and year 3 (N = 27 vaccine, 49 placebo) post-HIV-1 diagnosis. Composite scores are the geometric mean of fold over background per antigen consisting of 9 gp120s, 23 gp140s, 6 V1V2 gp70, and 4 V3 gp70 antigens. p values were calculated by Mann-Whitney test and adjusted by False Discovery Rate (FDR) for multiple comparisons. Only significant (p<0.05) p values are shown. (TIFF) [file ppat.1009101.s007.tiff]

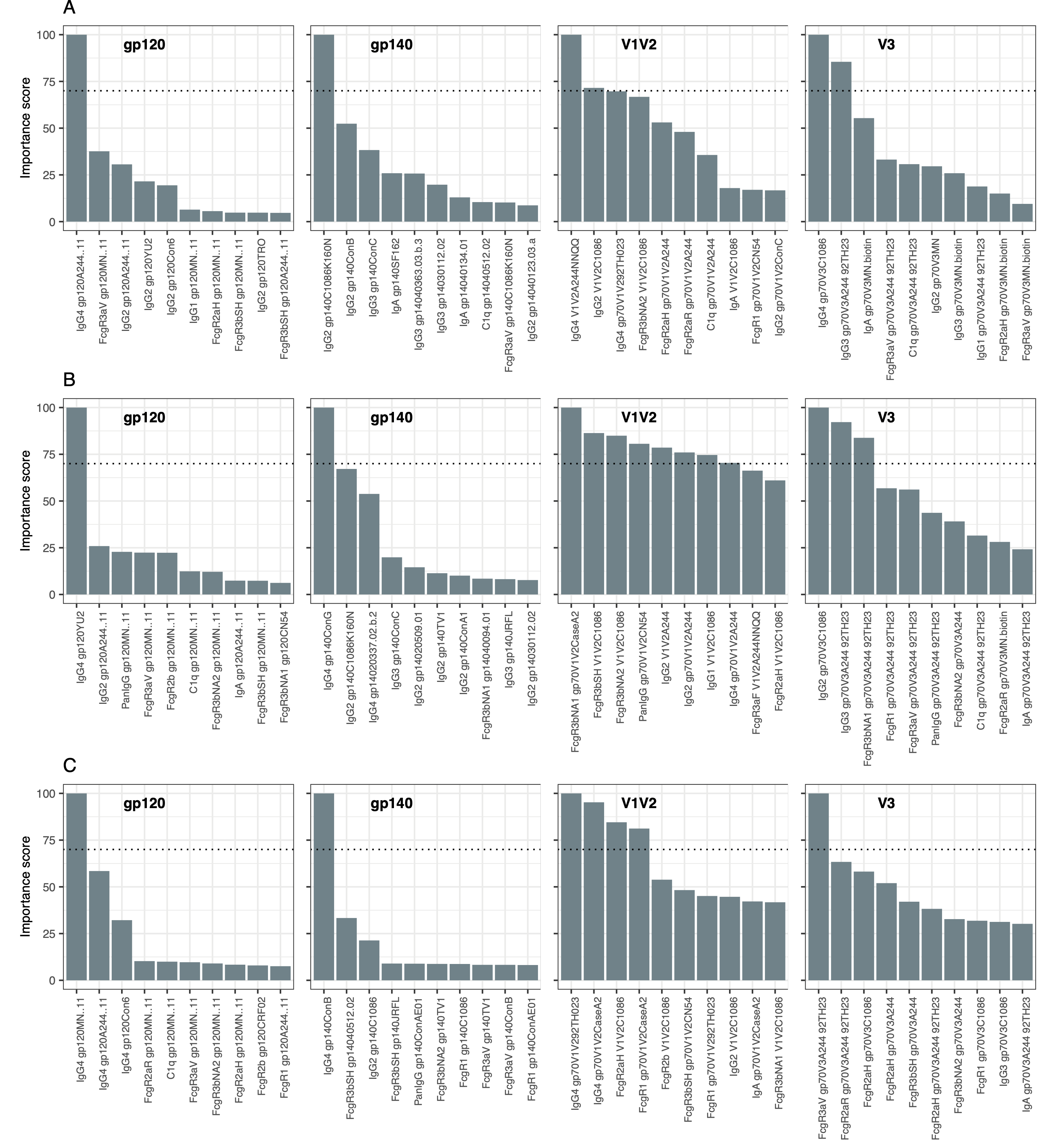

Supplement: S3 Fig — Binding features ranked by the average importance score of each variable at (A) 6 months, (B) year 1 and (C) year 3. Binding features are considered separately for each time point and HIV-1 antigen. Variables with a score above 70% (dashed line), corresponding to a 30% mean decrease in accuracy, are considered relevant. (TIFF) [file ppat.1009101.s008.tiff]

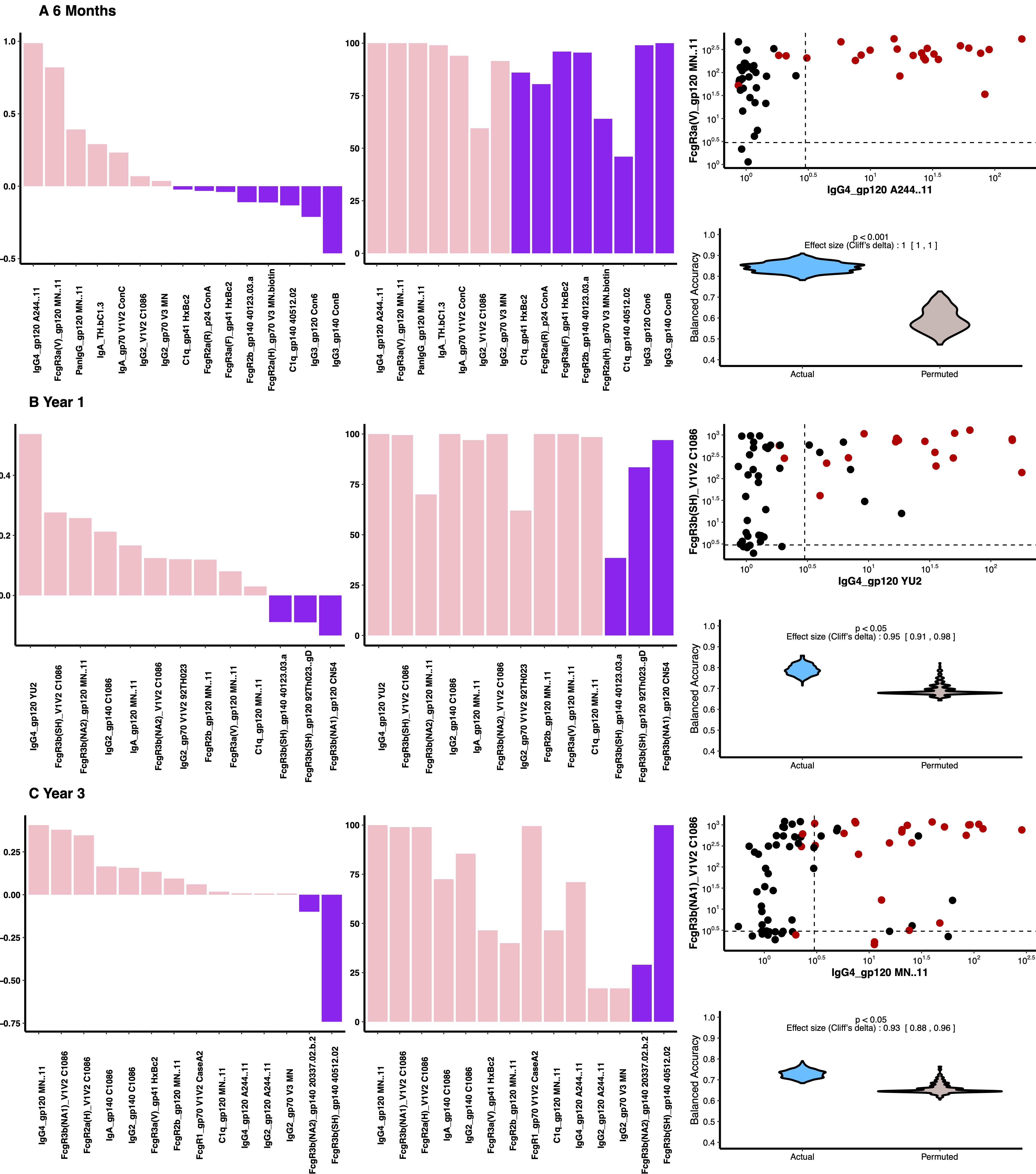

Supplement: S4 Fig — IgG4-gp120 responses and FcγR responses against V1V2 were robustly selected by LASSO to classify treatment arms. Left panel: coefficient weights for the final logistic regression model. Middle panel: percentage of each feature across CV folds and replicates for the features selected in the final logistic regression model. Right panel, top plot: visualization of the top two logistic regression coefficients by magnitude; vaccine recipients are represented in red and placebo recipients in black. Right panel, bottom plot: performance of the logistic regression classification from repeated cross-validation using actual versus permutated data with a one-sided P-value and for 200 independent repetitions; the effect size measured with Cliff’s delta shown in the figure. (TIFF) [file ppat.1009101.s009.tiff]

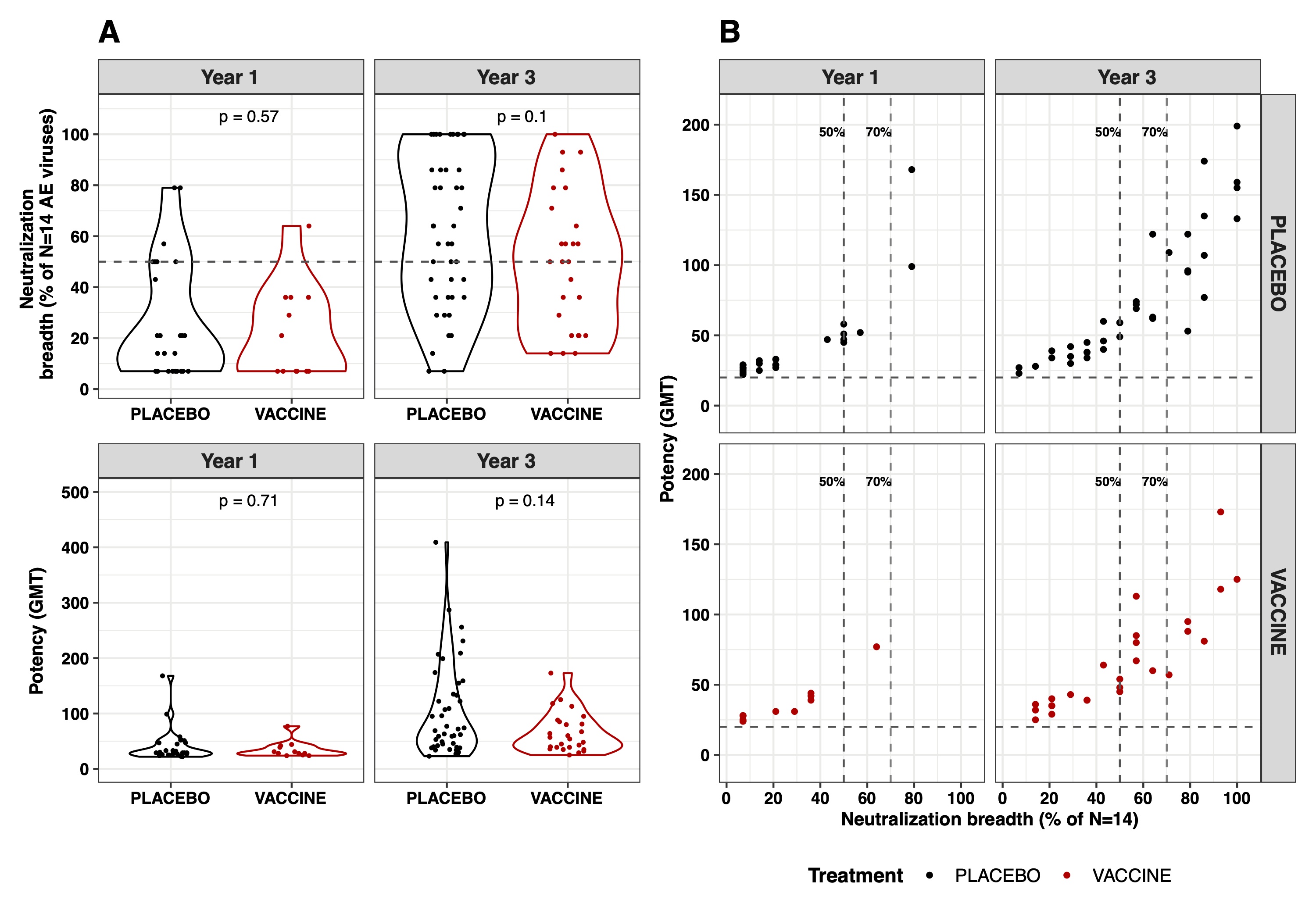

Supplement: S5 Fig — (A) Neutralization breadth (top) and potency (bottom) of vaccine (in red) and placebo (in black) recipients using the panel of 14 AE pseudoviruses compared in aggregate using Mann-Whitney t tests. (B) Spearman correlations of neutralization breadth and potency (geometric mean titer (GMT)) of placebo (top) and vaccine (bottom) recipients at year 1 (N = 12 vaccine, 25 placebo), and 3 (N = 26 vaccine, 43 placebo), post-diagnosis. Neutralization breadth is the percentage of viruses neutralized out of a panel of 14 CRF01_AE pseudoviruses. (TIFF) [file ppat.1009101.s010.tiff]

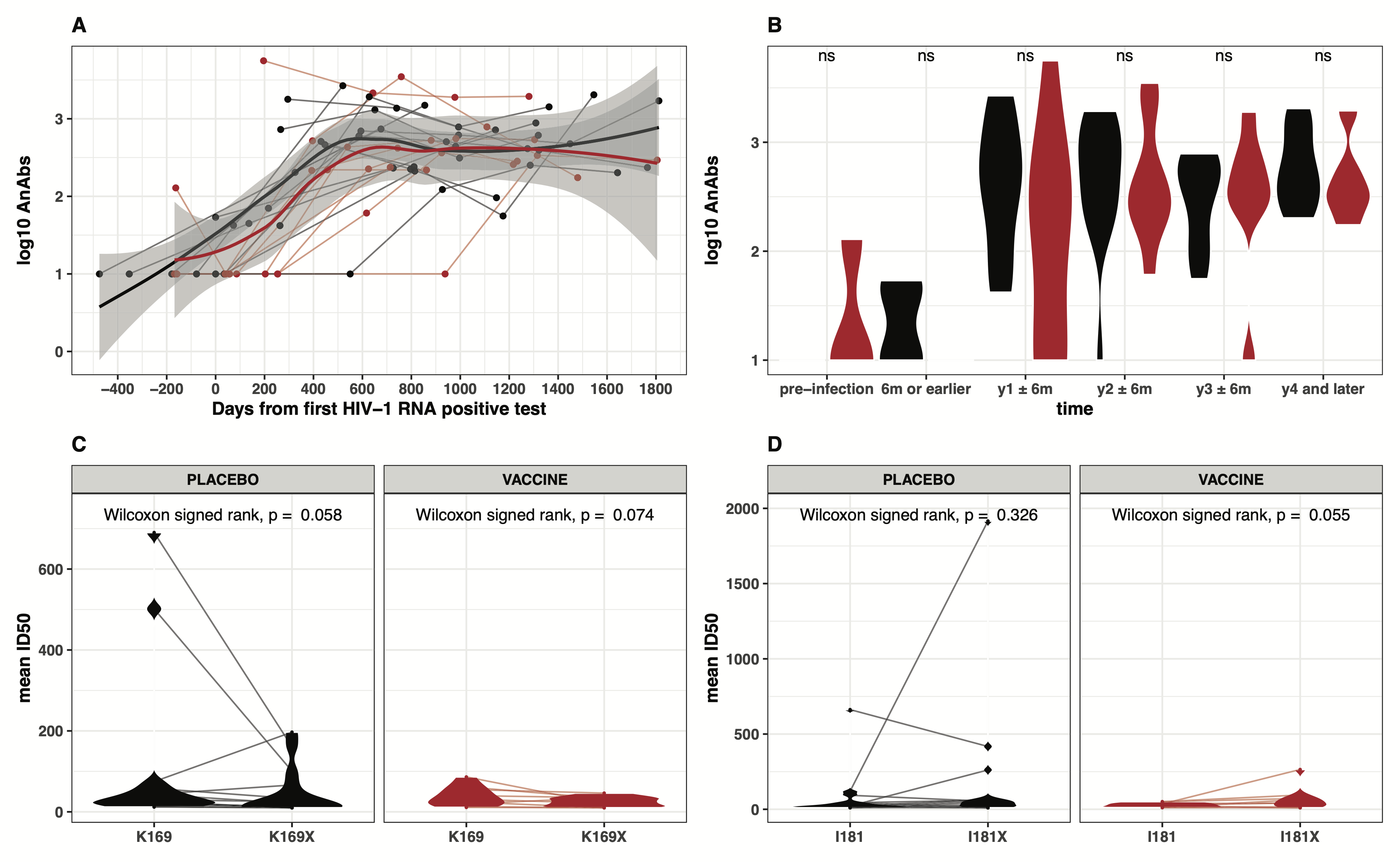

Supplement: S6 Fig — (A-B) Longitudinal autologous neutralization in vaccine (N = 9, red) and placebo (N = 14, black) recipients following HIV-1 diagnosis. (C-D) Neutralization responses against envelopes with or without residues associated with vaccine efficacy in RV144 (responses were measured in a matrix format against viruses from the 23 participants with available pseudoviruses). The HIV-1 Env variants associated with vaccine efficacy were K169 and I181X. (TIFF) [file ppat.1009101.s011.tiff]

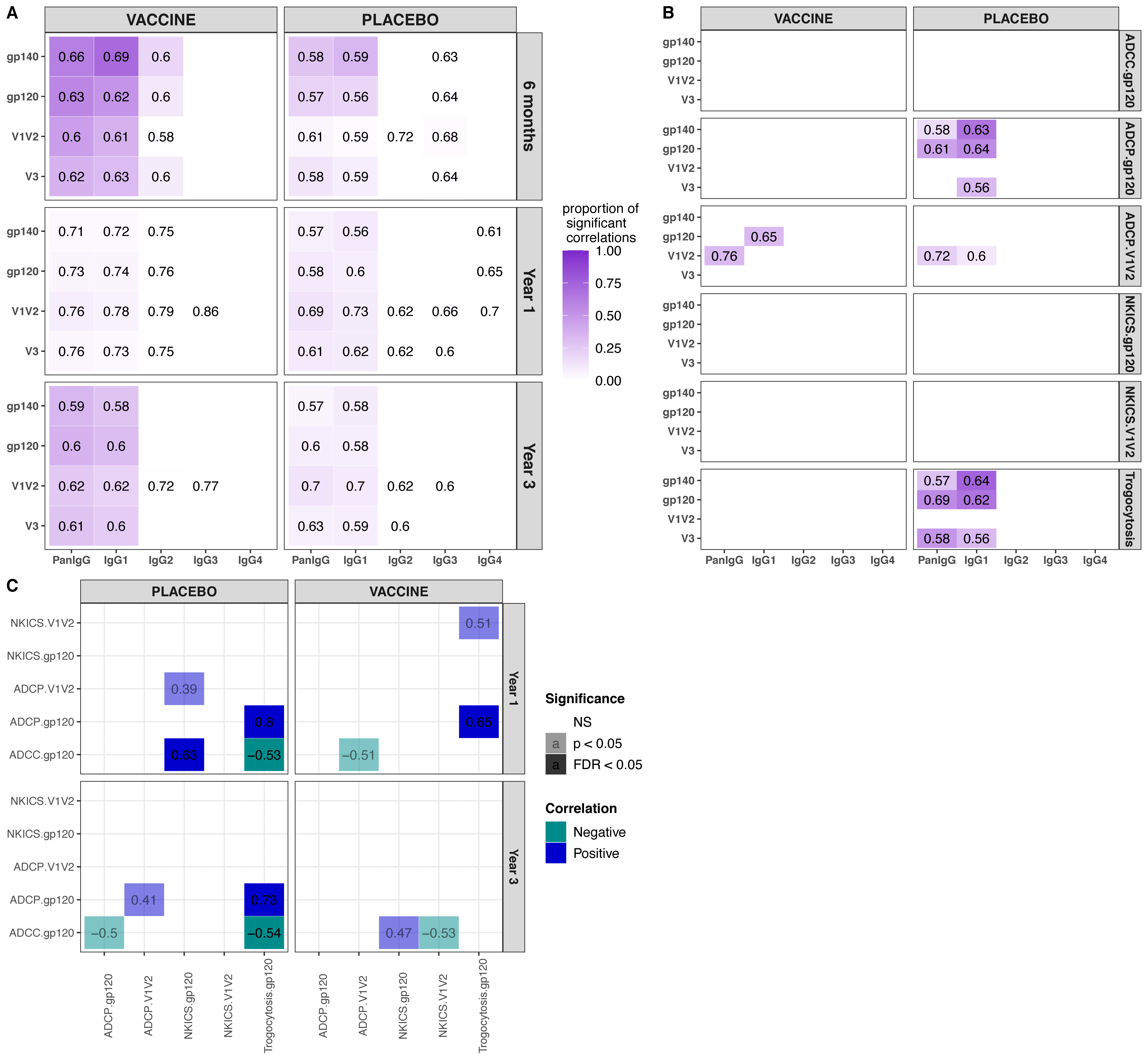

Supplement: S7 Fig — Panels A and B show the proportion of significant correlations (Spearman Rho > 0.5, p < 0.5) between IgG responses and either FcγR responses (A) or Fc effector function (B). The color represents the proportion of significant associations whereby higher proportions are marked in dark purple. The median Rho value of these significant correlations across all FcγR features is reported in each cell. (C) Significant Spearman correlations between Fc effector functions within each treatment groups. Blue: positive correlation; Cyan: negative correlation. Stronger colors represent significant correlations after correction for multiple testing. (TIF) [file ppat.1009101.s012.tif]

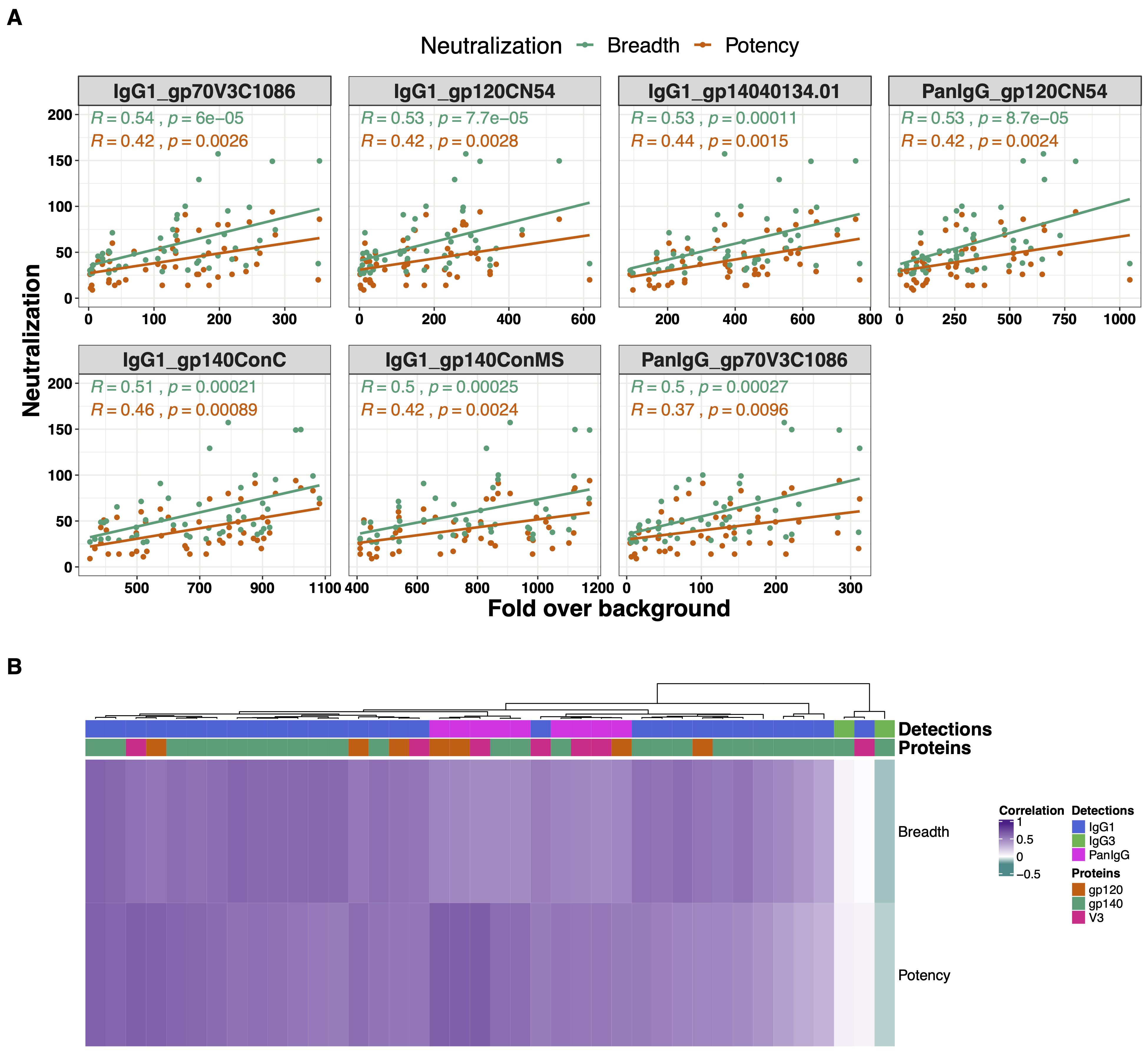

Supplement: S8 Fig — (A) Ig features associated with neutralization breadth or potency with Spearman Rho ≥ 0.5 (p-value ≤ 0.05). (B) Canonical sPLS model highlighted Ab binding features which covaried with the neutralization data in the placebo group at year 3 post-diagnosis. (TIFF) [file ppat.1009101.s013.tiff]

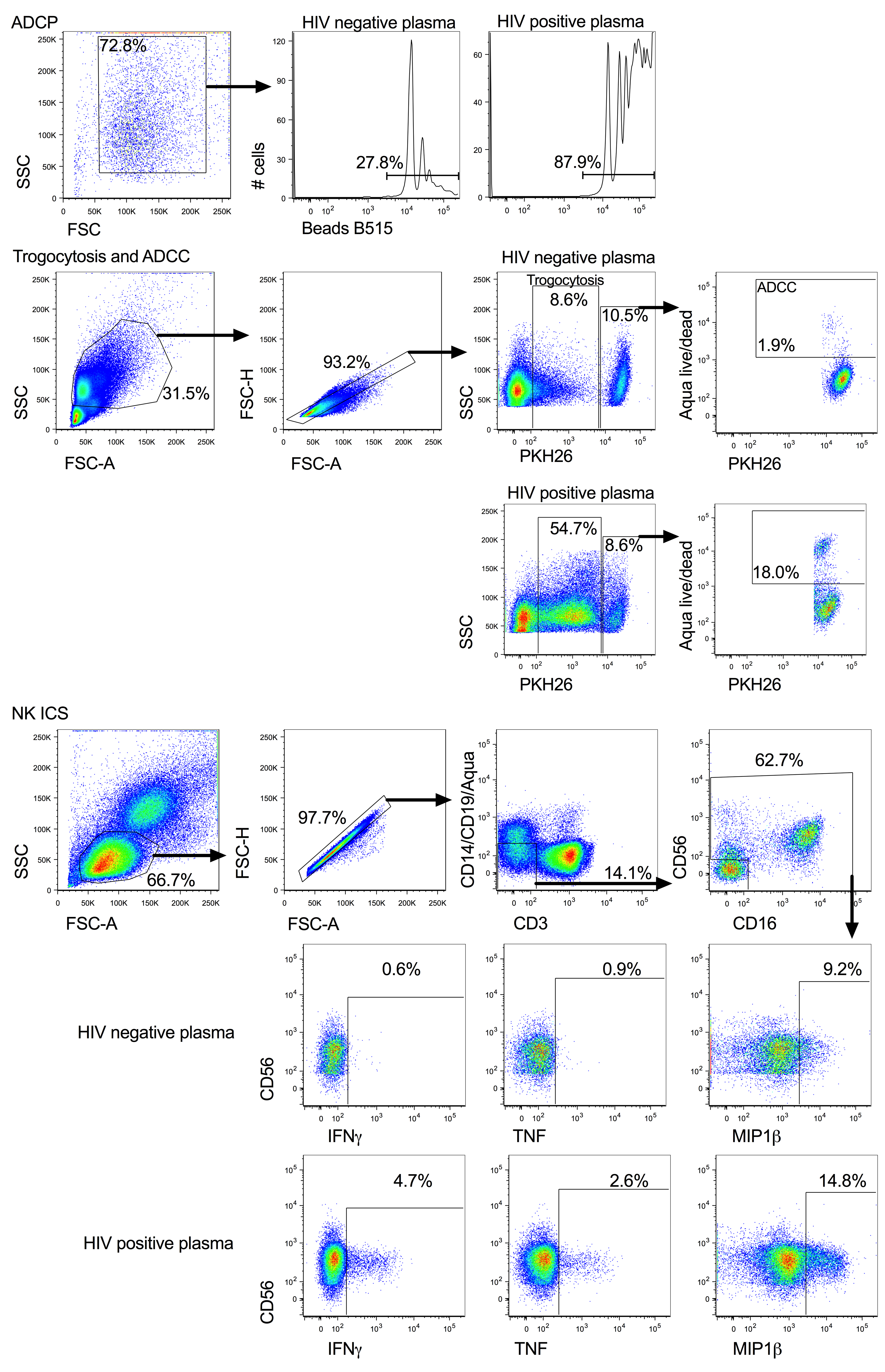

Supplement: S9 Fig — The gating strategy and representative results for one positive and negative control are shown for ADCP, trogocytosis, ADCC, and NK cell activation. (TIFF) [file ppat.1009101.s014.tiff]
